# Supplementary material for: Binless normalization of Hi-C data provides significant interaction and difference detection independent of resolution
Source: Nat Commun. 2019 Apr 26;10:1938. doi: 10.1038/s41467-019-09907-2 (PMC6486590; doi:10.1038/s41467-019-09907-2)
Supplement: Supplementary file 1 — Supplementary Information [file 41467_2019_9907_MOESM1_ESM.pdf]

Supplementary Information for: “Binless normalization of Hi-C data provides significant interaction and difference detection independent of resolution”

Spill *et al.*

Table of contents

Supplementary Figures : page 2
Supplementary Methods : page 31
Supplementary References : page 46

Supplementary Figures

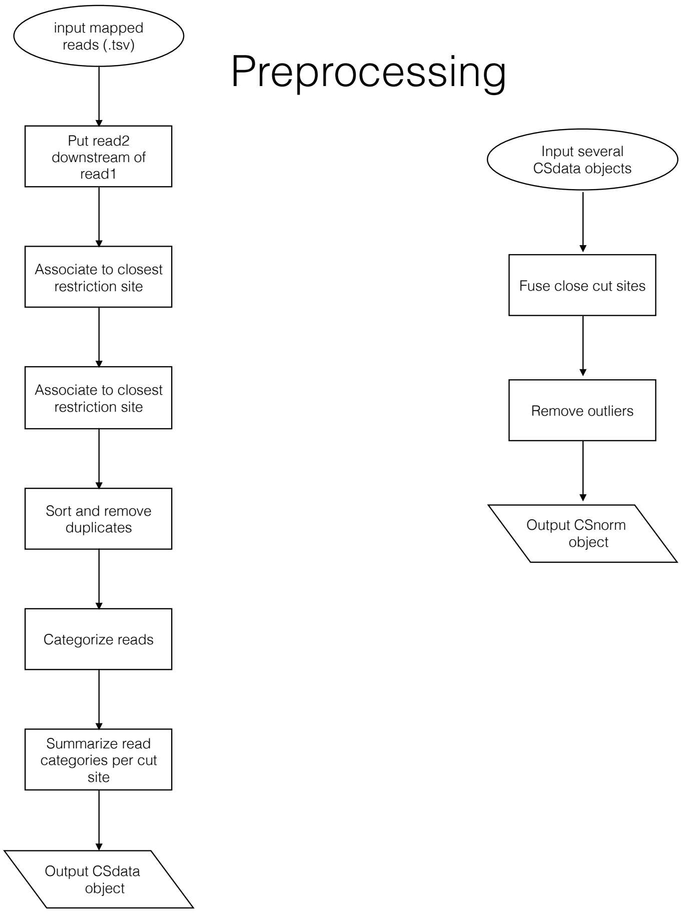

Supplementary Figure 1a. Flowchart for binless preprocessing

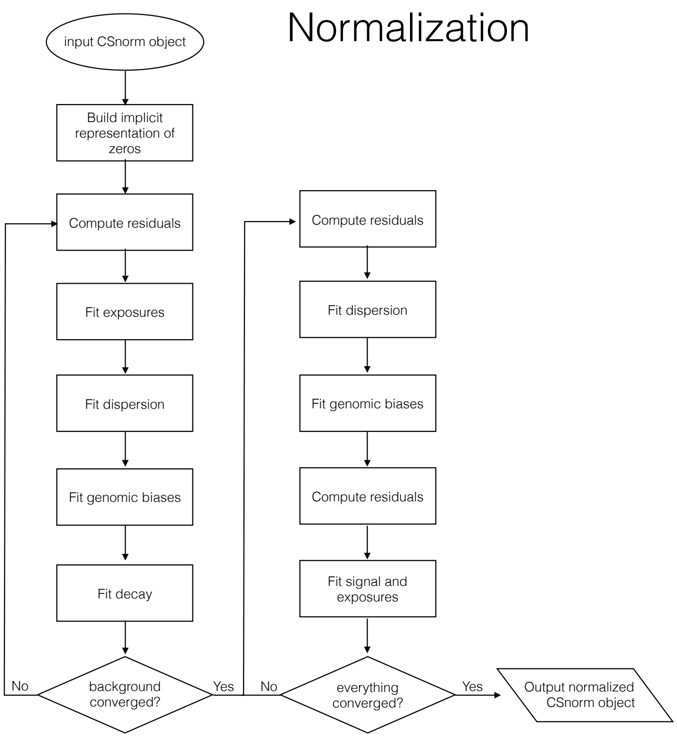

Supplementary Figure 1b. Flowchart for binless normalization

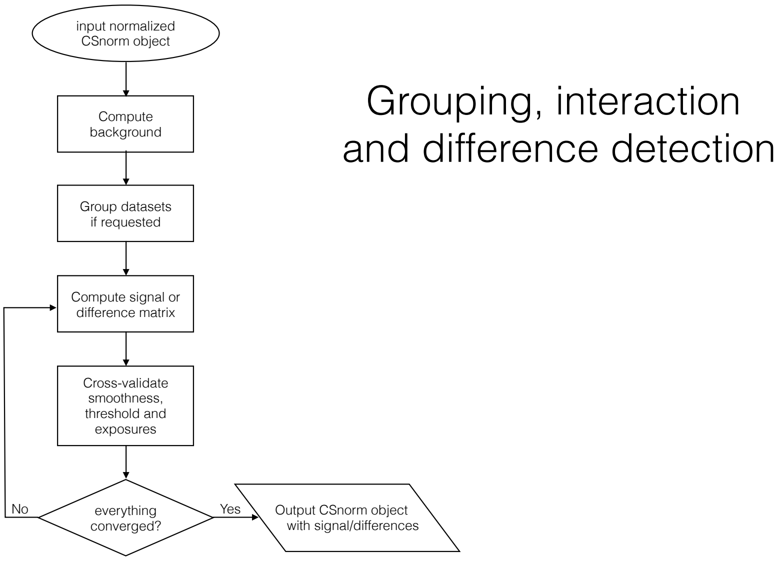

Supplementary Figure 1c. Flowchart for binless interaction detection

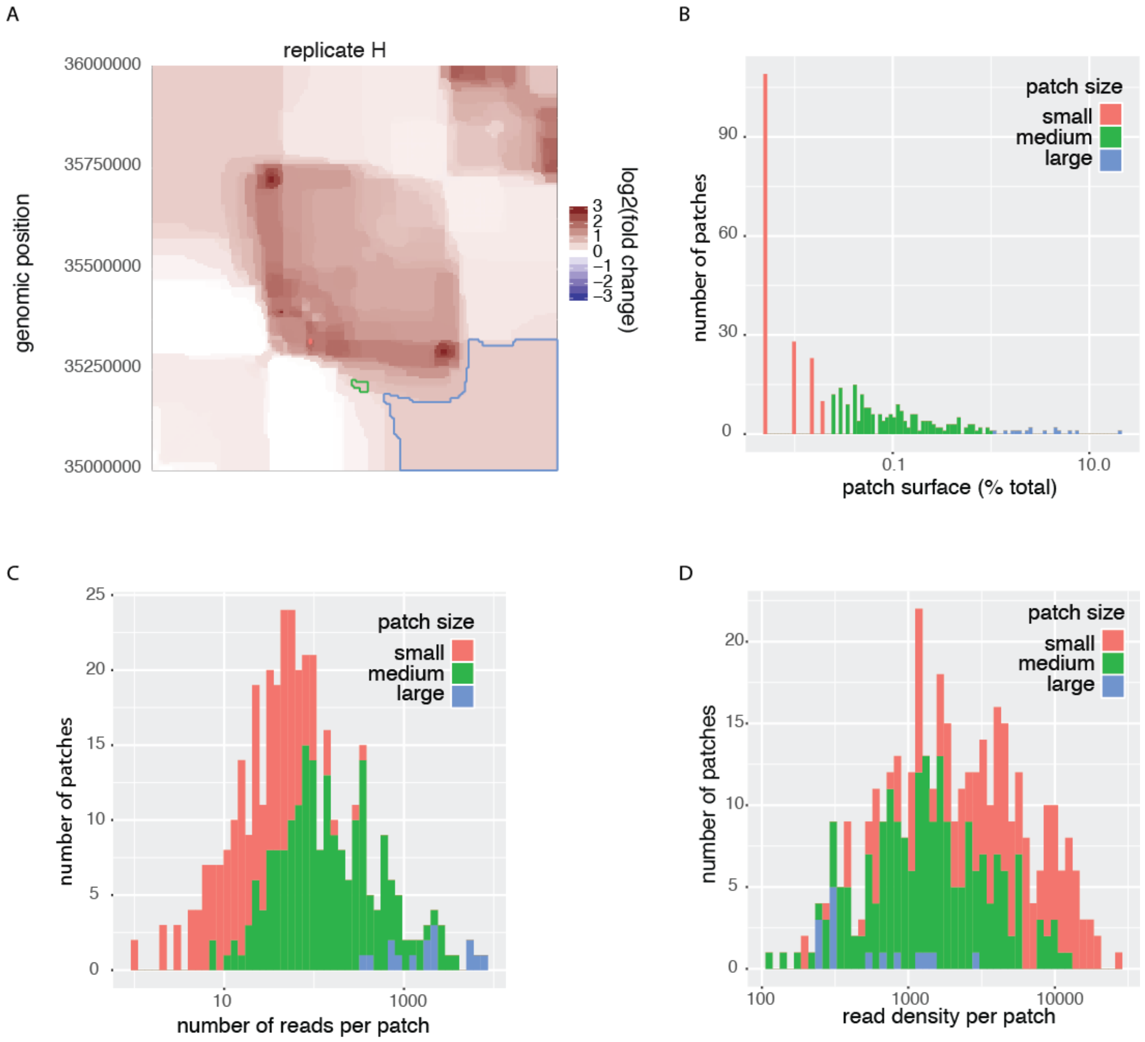

Supplementary Figure 2. Patch statistics in the binless matrix of a 1Mb example region (HIC003, hg19 [4], called "Replicate H" in [34]). A) binless signal matrix of the region, with three patches of varying sizes highlighted. B) Distribution of patch sizes in the binless matrix. Patches are grouped in three size categories for illustration. C) Distribution of the number of reads per patch. D) Distribution of the read density per patch.

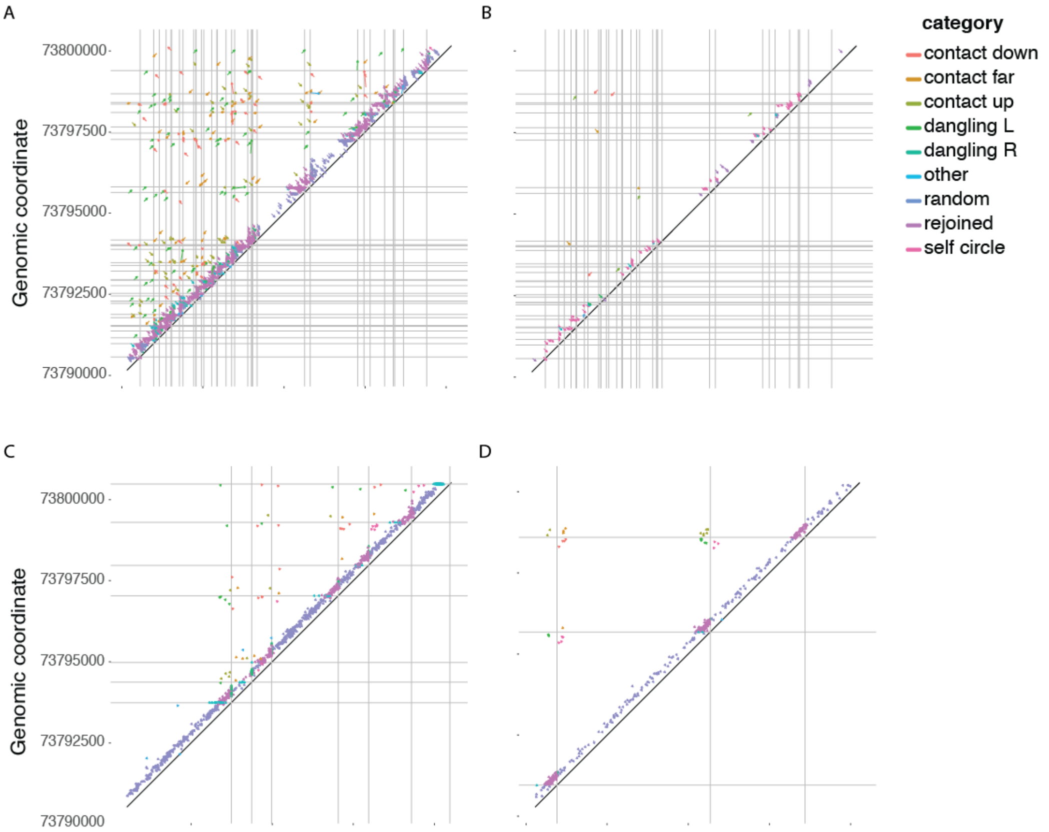

Supplementary Figure 3. Base-resolution representation of a 10kb region on the TSIX gene in four different datasets (data from [4]). A) MboI HIC001-HIC018 combined. B) MboI HIC006. C) HindIII HIC035. D) NcoI HIC036.

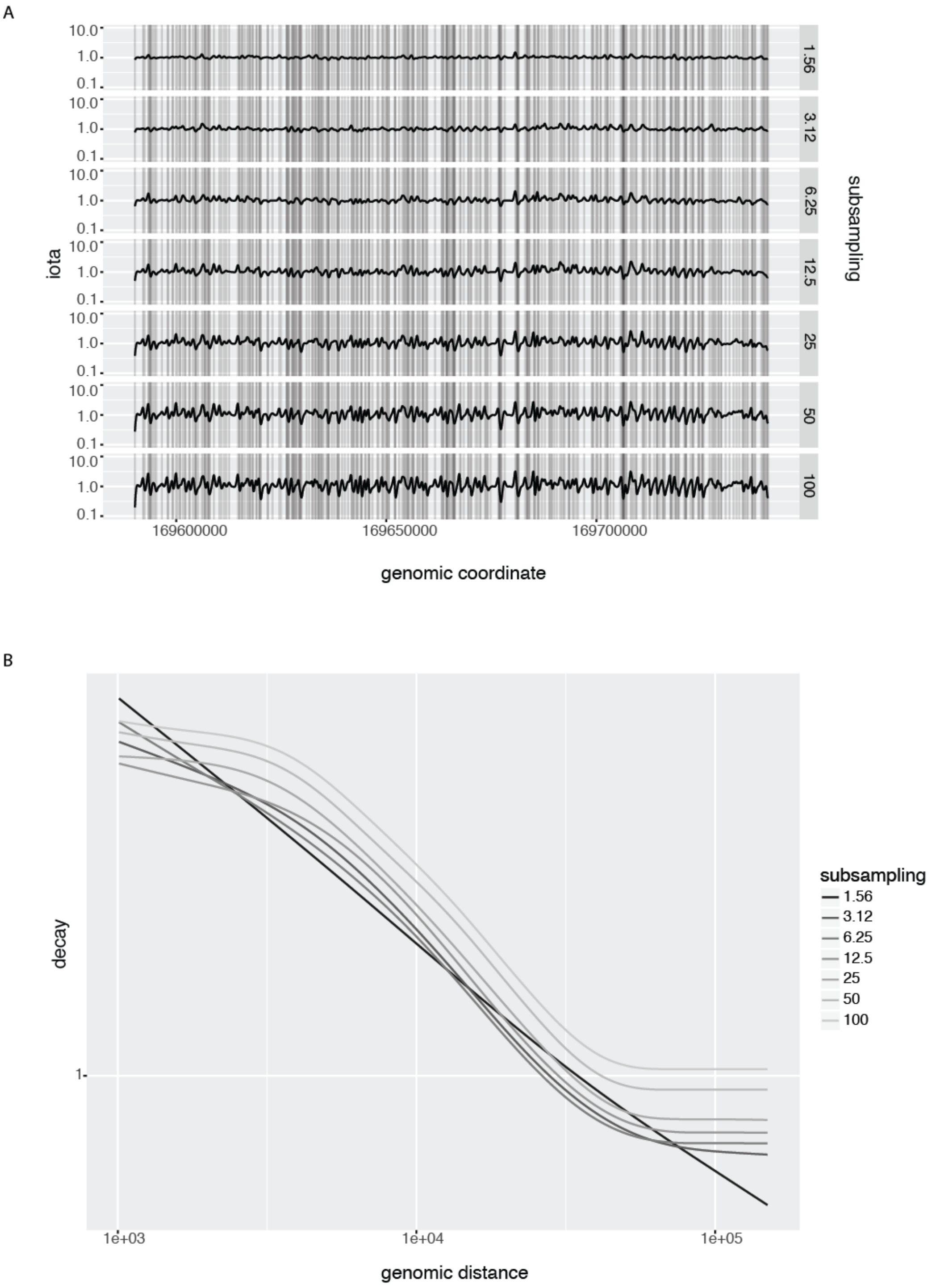

Supplementary Figure 4. Binless data smoothing for the SELP gene locus in the human chromosome 1 (data from [4])**.** A) ι genomic bias. Vertical lines are cut site locations. Right labels are percent of reads used for normalization. Smaller quantities of data lead to a smoother bias, which indicates that Generalized Additive Models do not overfit the data and can be used for normalization of Hi-C experiments. B) The same trend is observable in the diagonal decay.

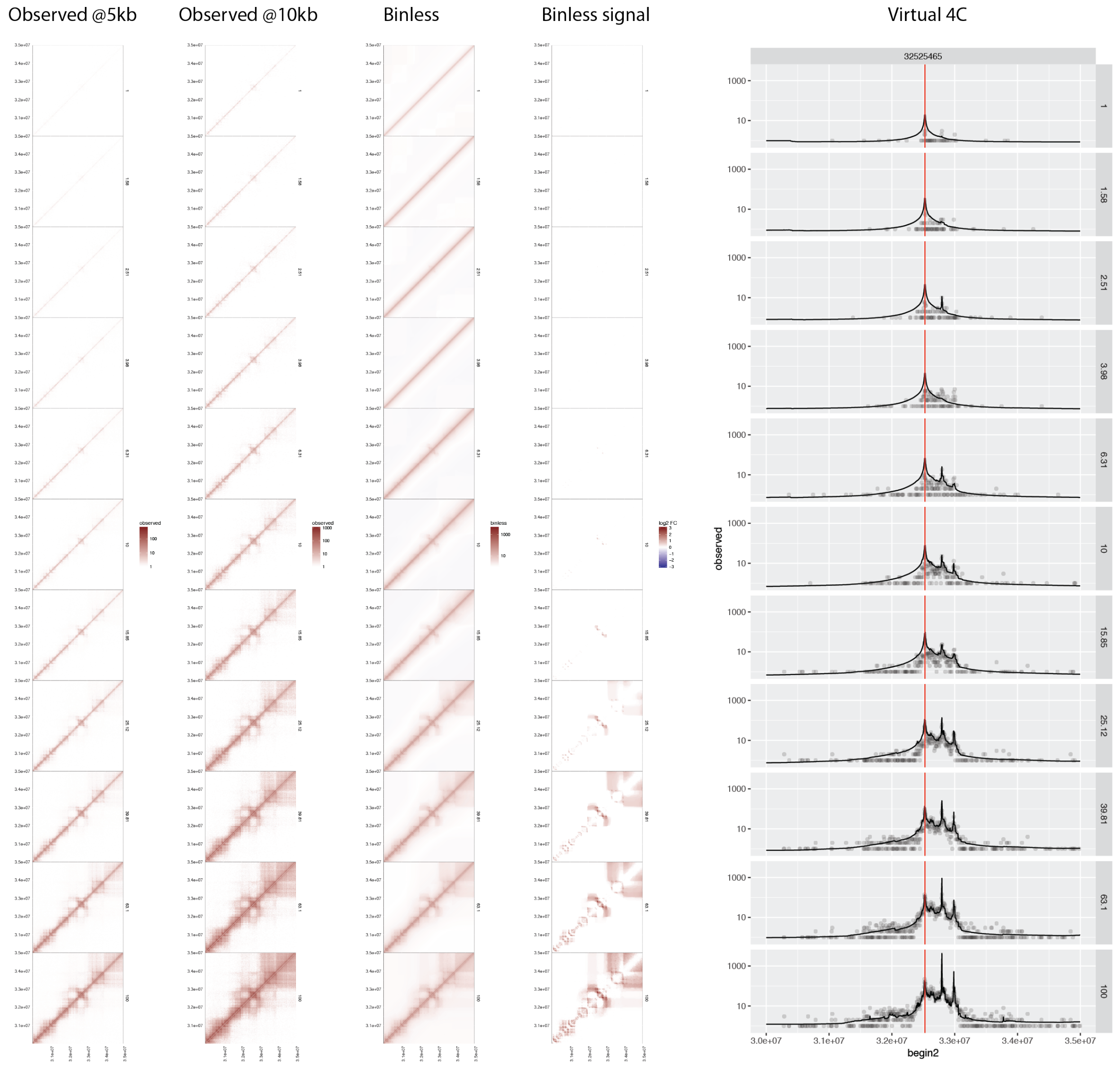

Supplementary Figure 5. Effect of subsampling on binless matrices. Zoom on the 5Mb region chr22:30000000-35000000 of the combined data from all IMR90 replicates of Rao *et al* (HIC050 to HIC056). Between 1% and 100% of the data was subsampled randomly and normalized using *Binless* on the whole chromosome 22 at 5kb base resolution. From left to right: Raw data at 5kb and 10kb resolution, binless matrix, signal matrix and virtual 4C on a 5kb viewpoint at chr22:32525465-32530465. For this last plot, observed data is dotted, and solid lines correspond to the binless matrix row.

A

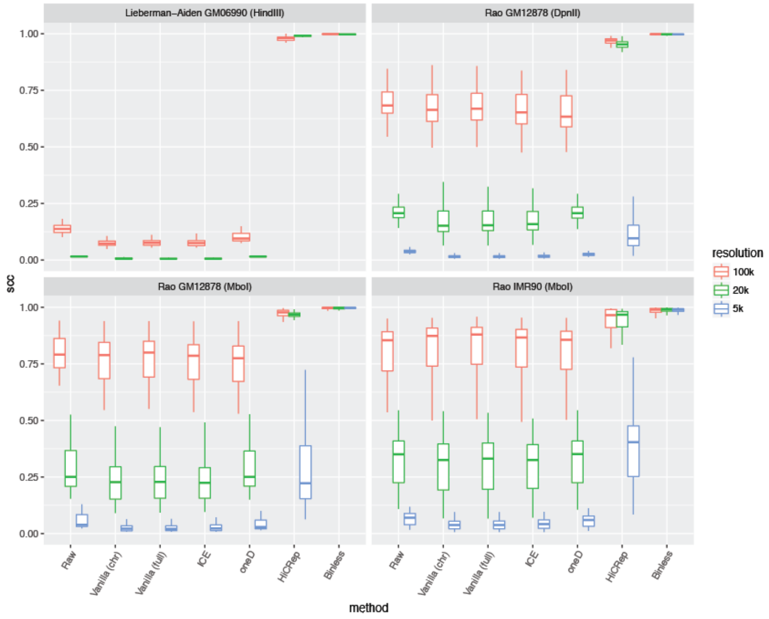

B

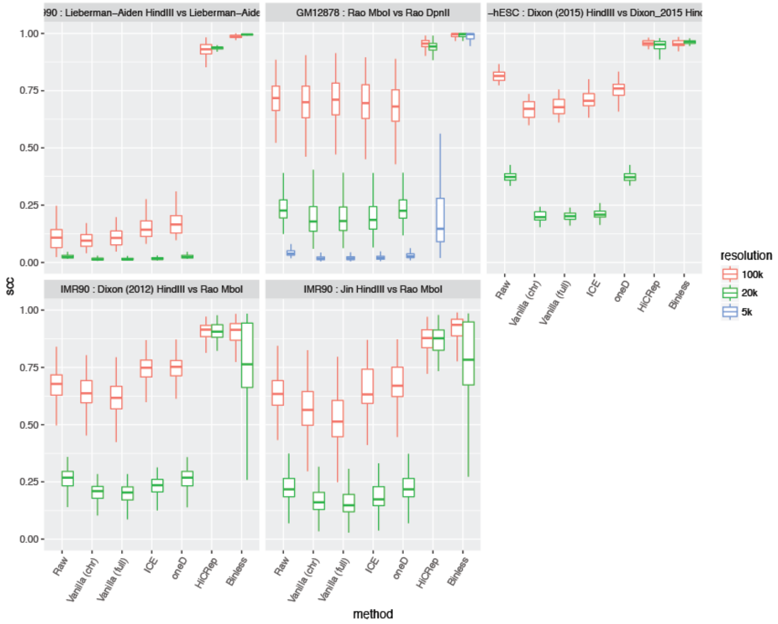

Supplementary Figure 6. Binless matrices lead to increased reproducibility in Hi-C matrices at all resolutions. A, B: stratified correlation coefficient. A: technical replicates, B: different enzymes. (see methods for details and Sup. Table panel 8 for sample sizes).

C

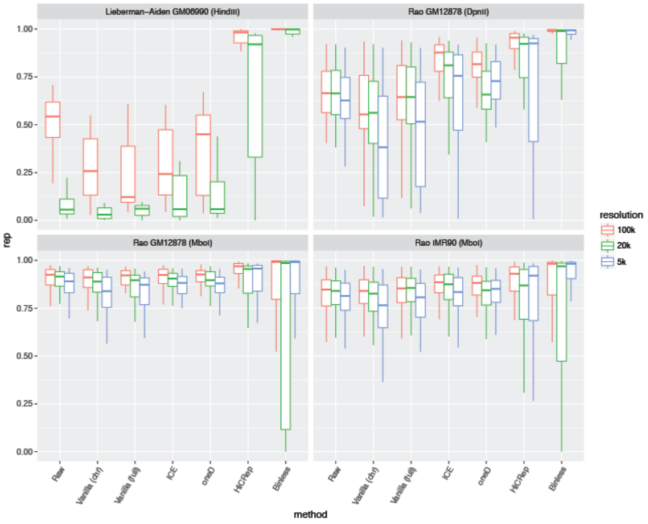

D

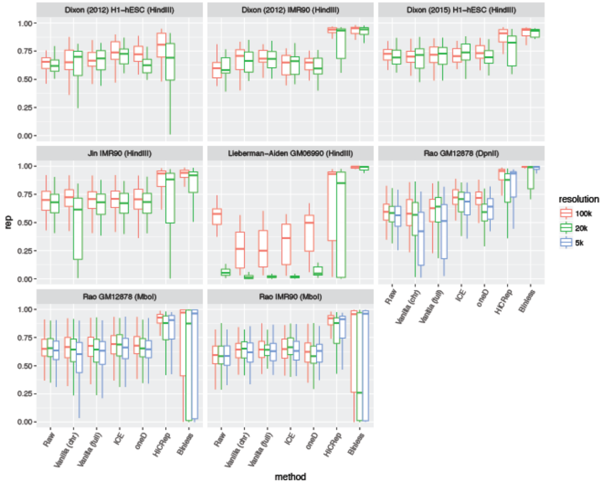

E

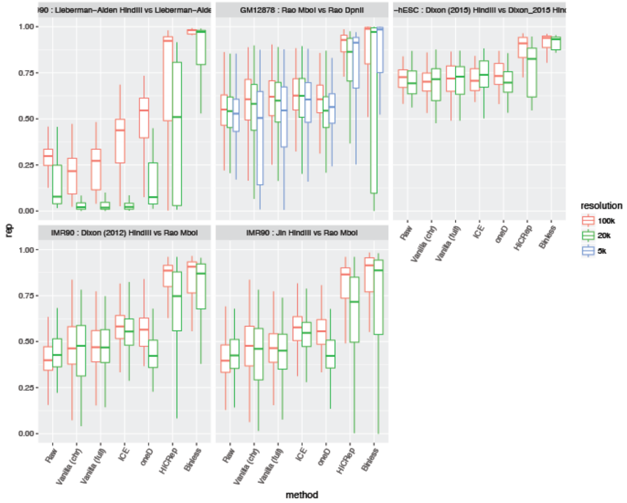

Supplementary Figure 6 (cont.) C, D, E: reproducibility index. C: technical replicates, D: biological replicates. E: different enzymes. (see methods for details and Sup. Table panel 8 for sample sizes).

F

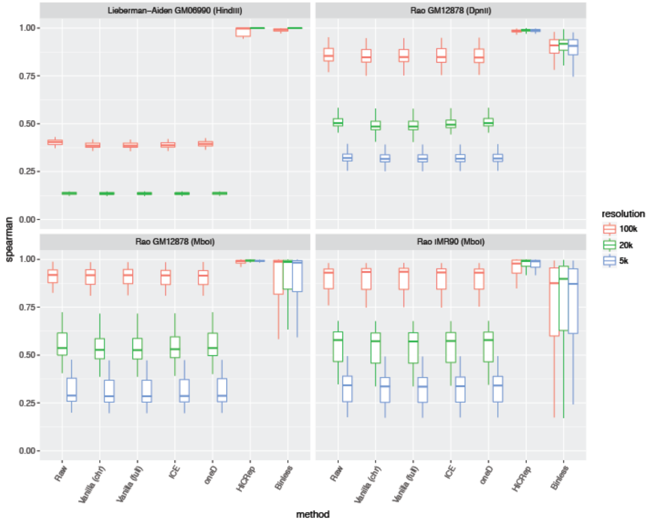

G

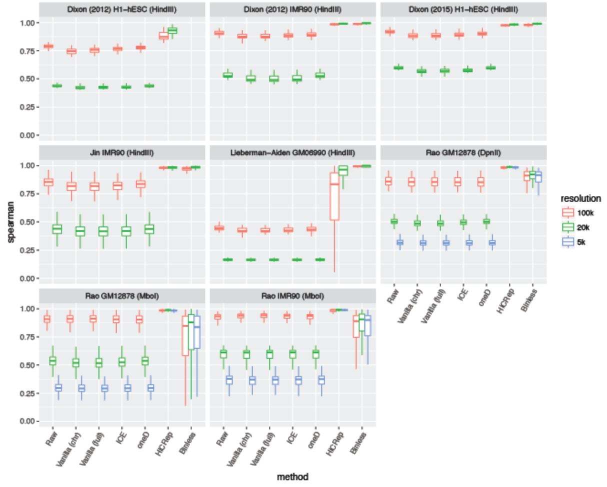

H

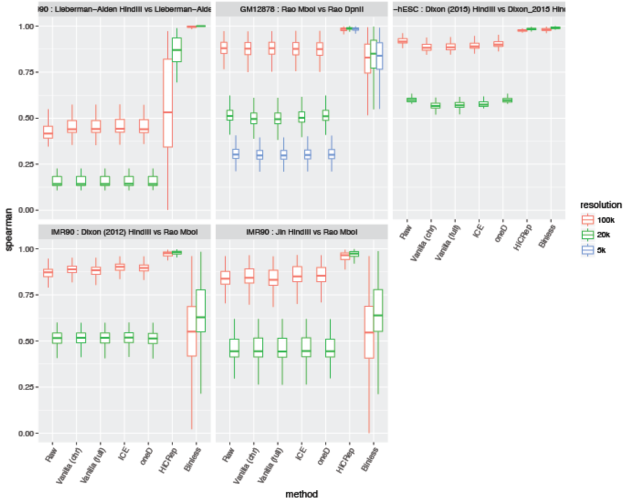

Supplementary Figure 6 (cont.) F, G, H: spearman correlation. F: technical replicates, G: biological replicates. H: different enzymes. (see methods for details and Sup. Table panel 8 for sample sizes).

I

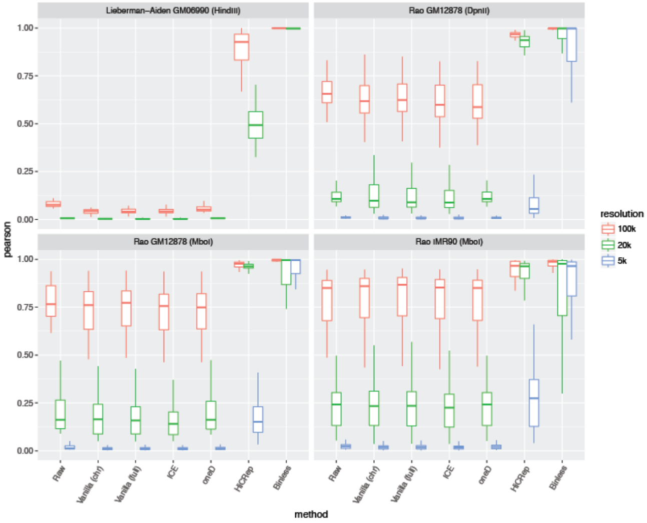

J

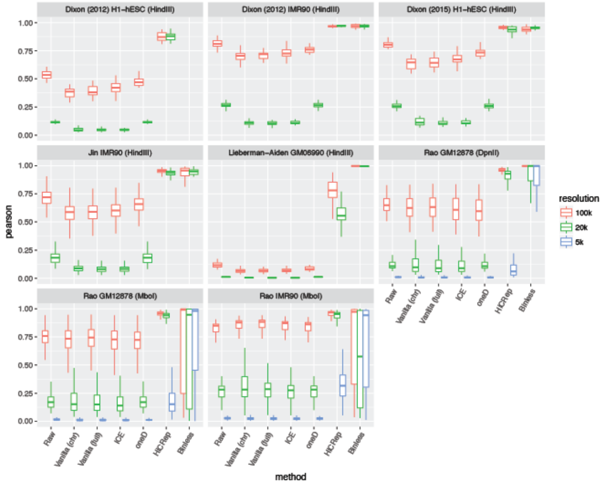

K

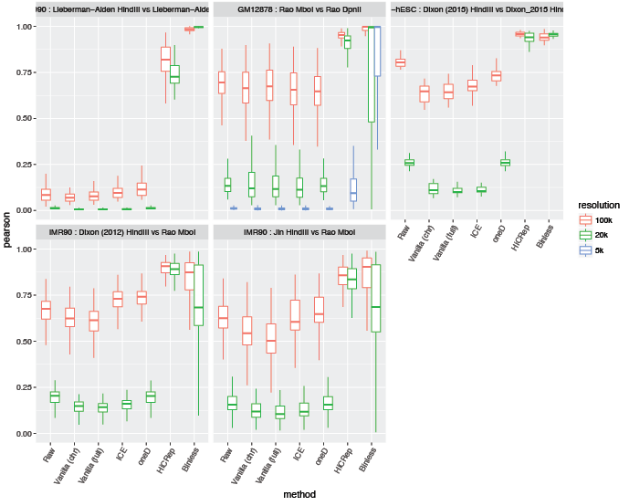

Supplementary Figure 6 (cont.) I, J, K: Pearson correlation (after background adjustment, see methods). I: technical replicates, J: biological replicates. K: different enzymes. (see methods for details and Sup. Table panel 8 for sample sizes).

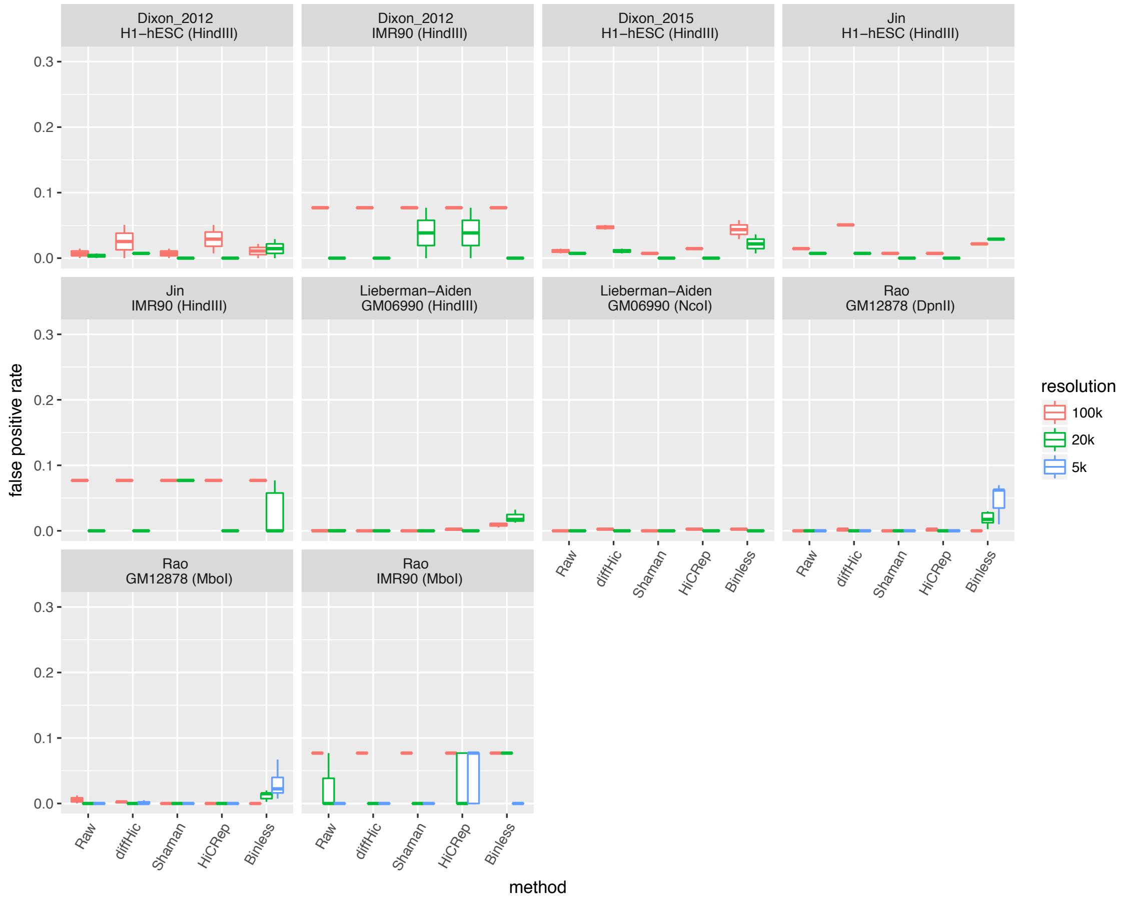

Supplementary Figure 7: False positive rate in Binless is maintained below 2.4% on average. Each box reports the false positive rate at several resolutions, for each interaction detection method. (see methods for details and Sup. Table panel 9 for total number of annotated true negatives)

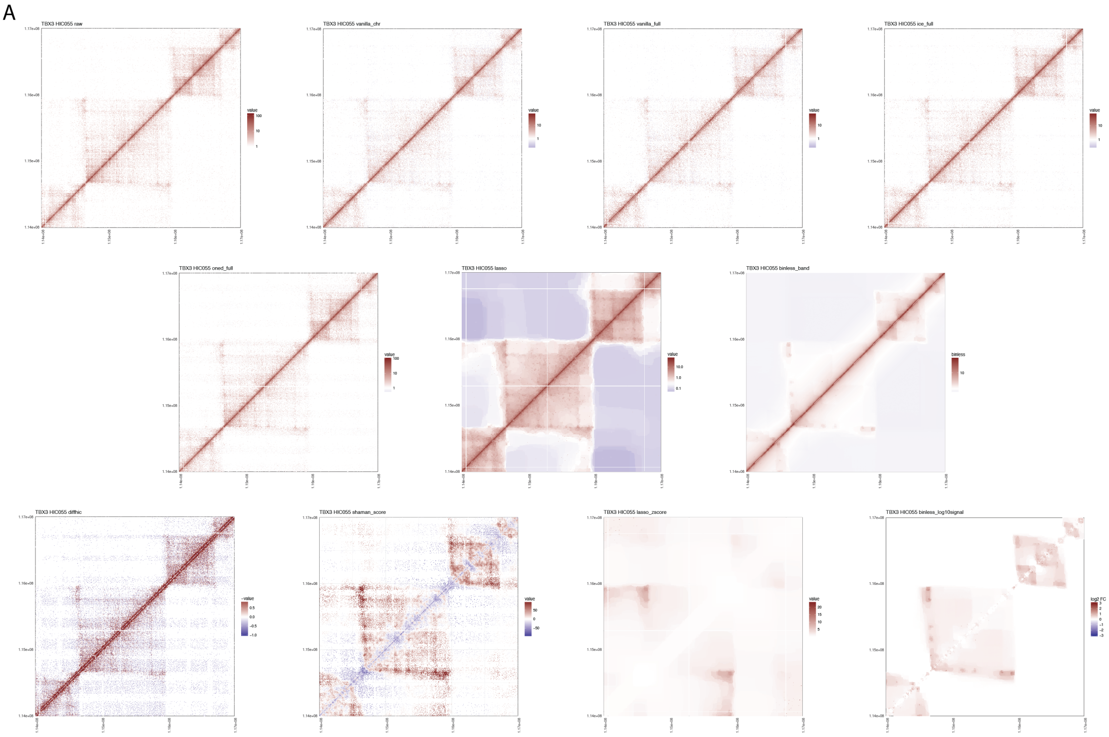

Supplementary Figure 8: Example loci for all produced outputs. A: TBX3 locus (Rao HIC055 chr12:114M-117M at 5kb resolution). Top row, from left to right: raw matrix, vanilla by chromosome, vanilla whole-genome, ICE whole-genome. Middle row: oneD, HiCRep, Binless. Bottom row: diffHic enrichment, shaman score, HiCRep z-score, Binless signal.

B

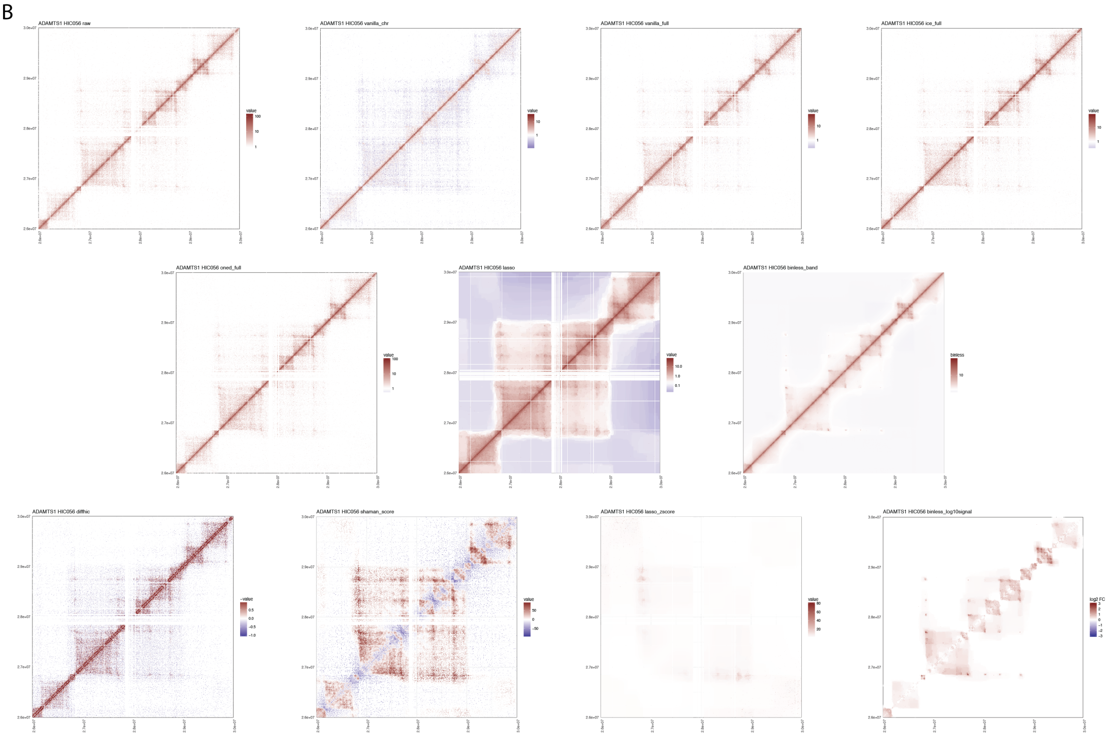

Supplementary Figure 8 (cont.) B: ADAMTS1 locus (Rao HIC056 chr21:26M-30M at 5kb resolution).

C

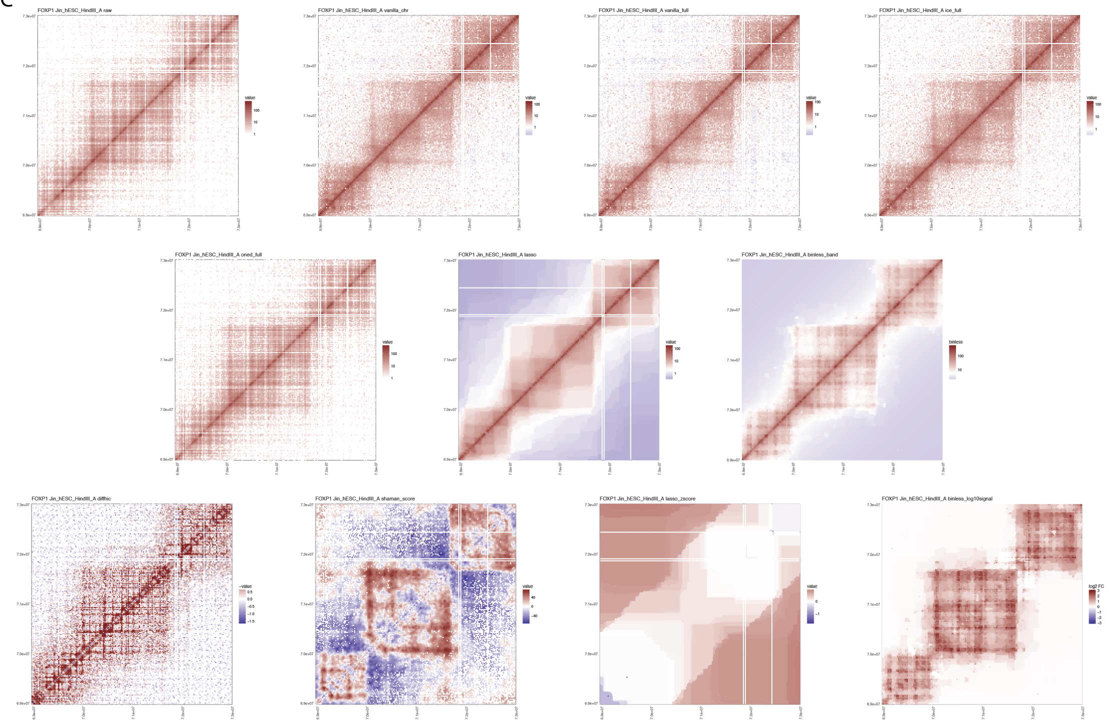

Supplementary Figure 8 (cont.) C: FOXP1 locus (Jin_hESC_HindIII_A chr3:69M-73M at 20kb resolution).

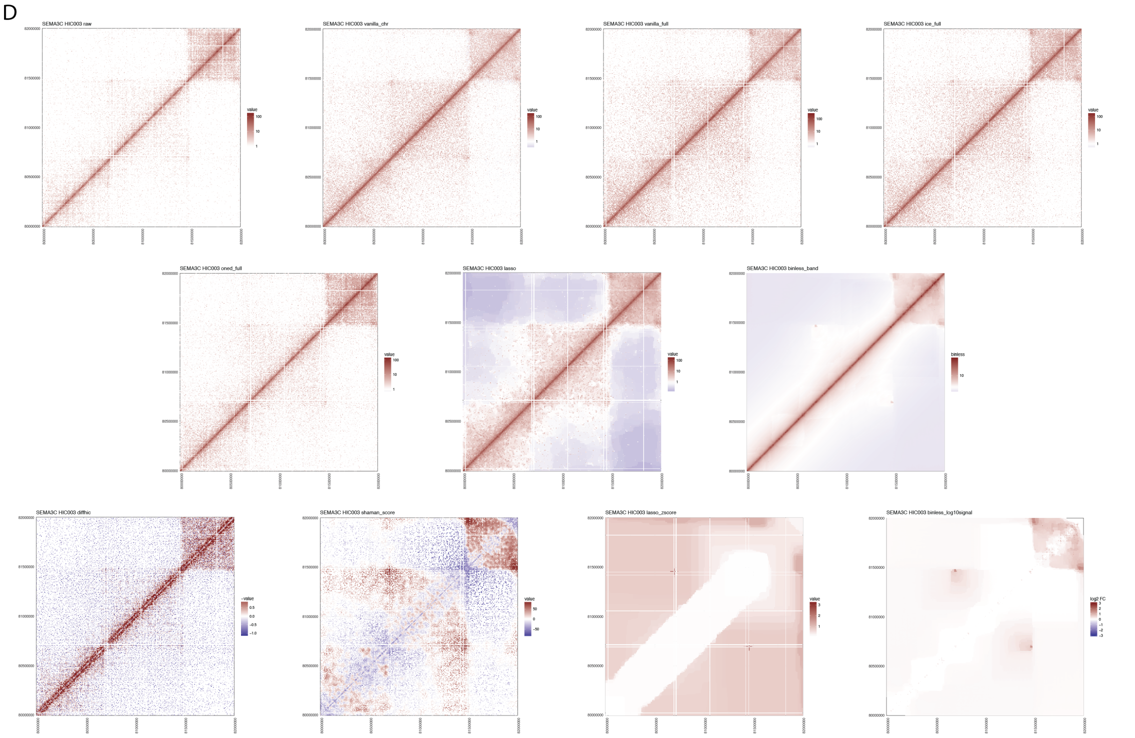

Supplementary Figure 8 (cont.) D: SEMA3C locus (Rao HIC003 chr7:80M-82M at 5kb resolution).

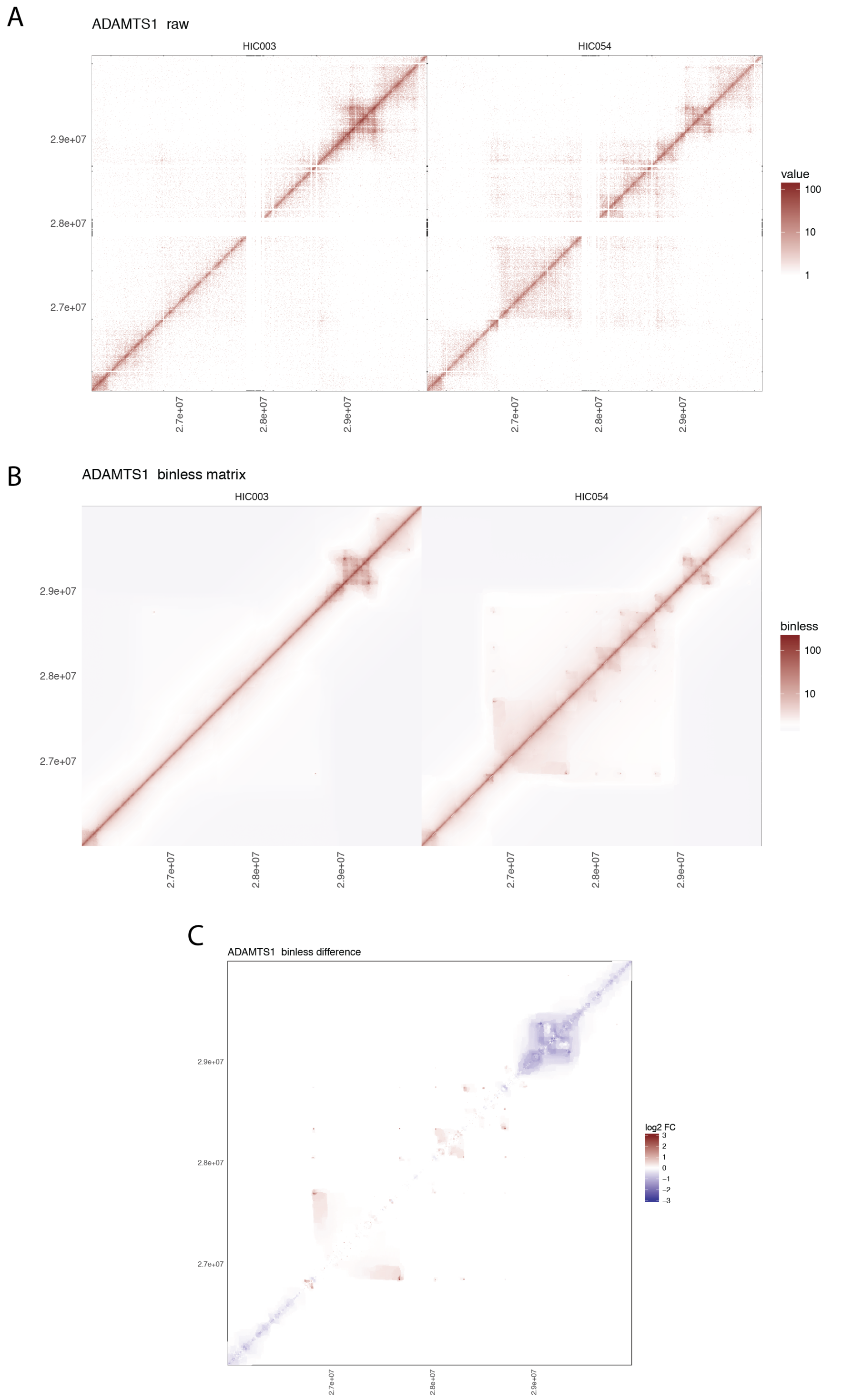

Supplementary Figure 9. Example of difference calculations with Binless. ADAMTS1 locus (chr21:26M-30M, Rao HIC003 vs HIC054). A: Raw data. B: binless matrix. C: binless difference matrix.

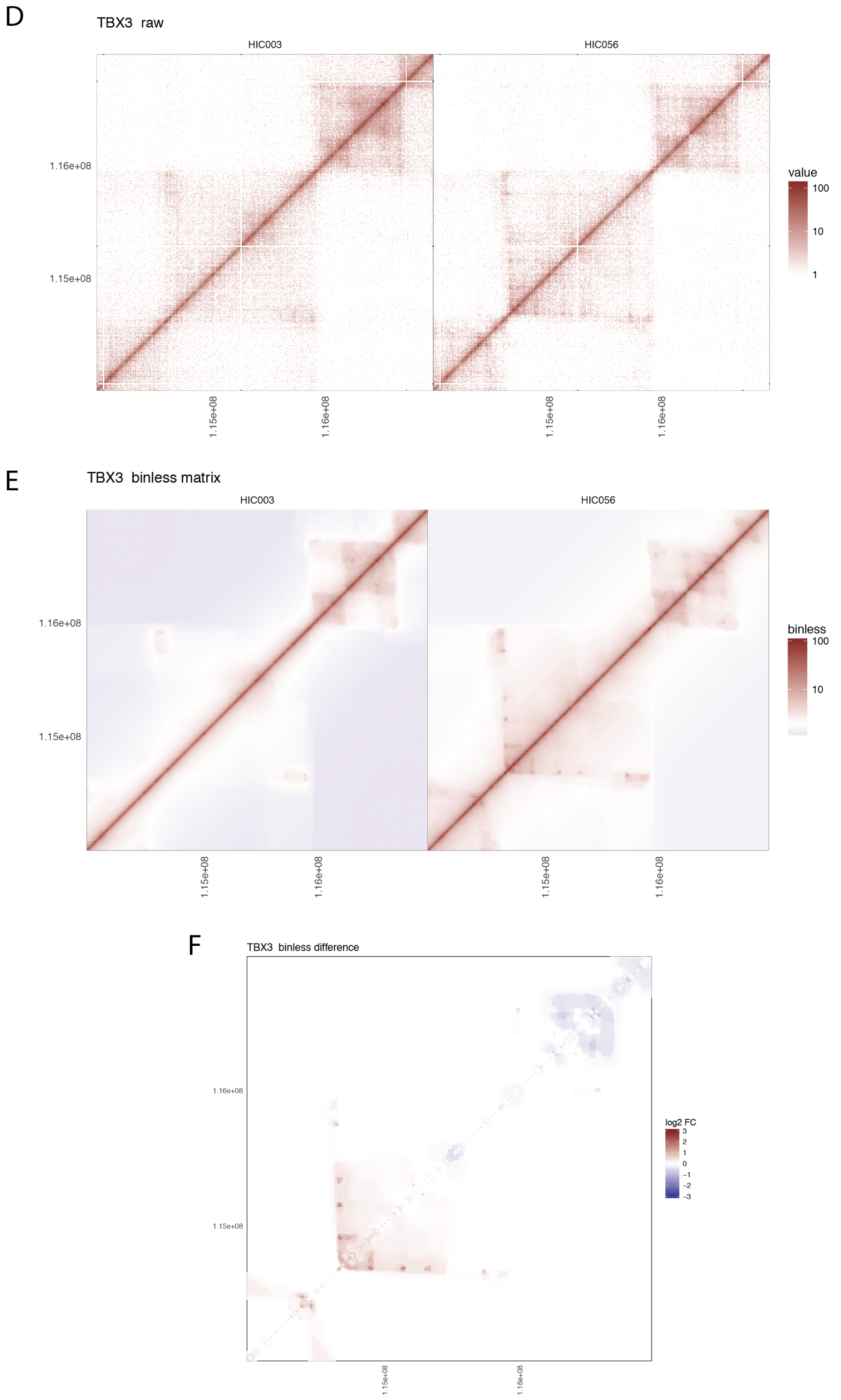

Supplementary Figure 9 (cont.). Example of difference calculations with Binless. TBX3 locus (chr12:114M-117M, Rao HIC003 vs HIC056). D: Raw data. E: binless matrix. F: binless difference matrix.

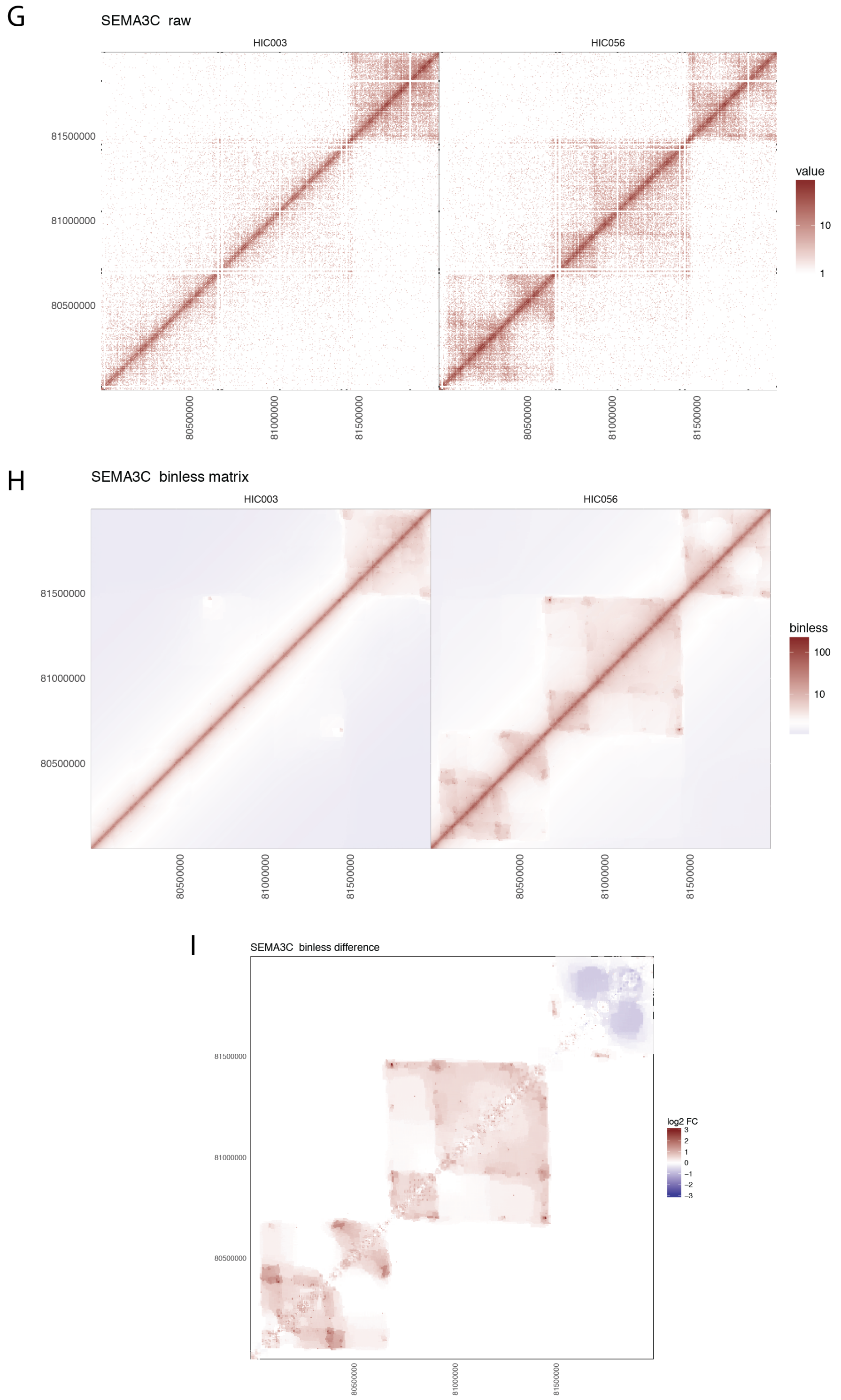

Supplementary Figure 9 (cont.). Example of difference calculations with Binless. SEMA3C locus (chr7:80M-82M, Rao HIC003 vs HIC056). G: Raw data. H: binless matrix. I: binless difference matrix.

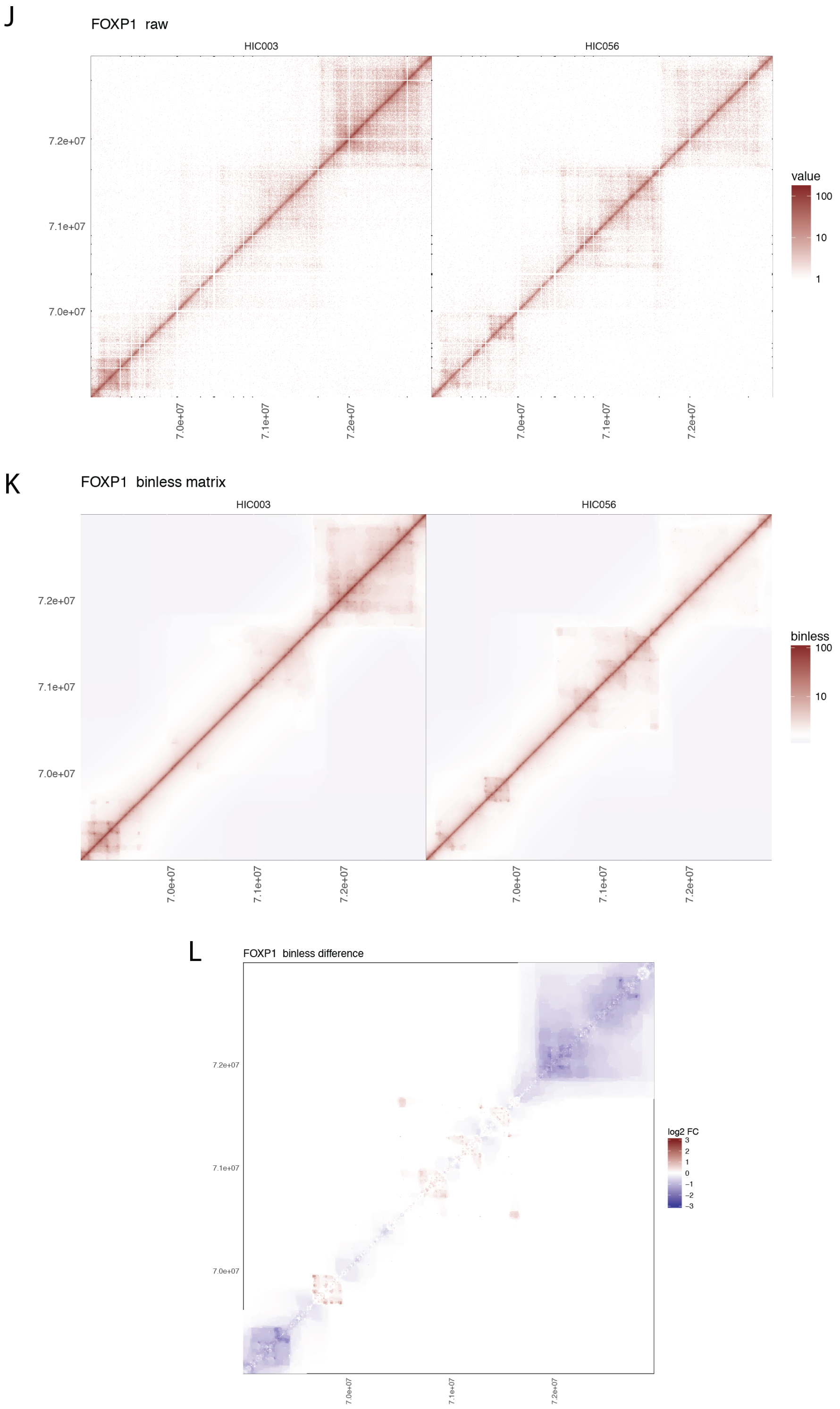

Supplementary Figure 9 (cont.). Example of difference calculations with Binless. FOXP1 locus (chr3:69M-73M, Rao HIC003 vs HIC056). J: Raw data. K: binless matrix. L: binless difference matrix.

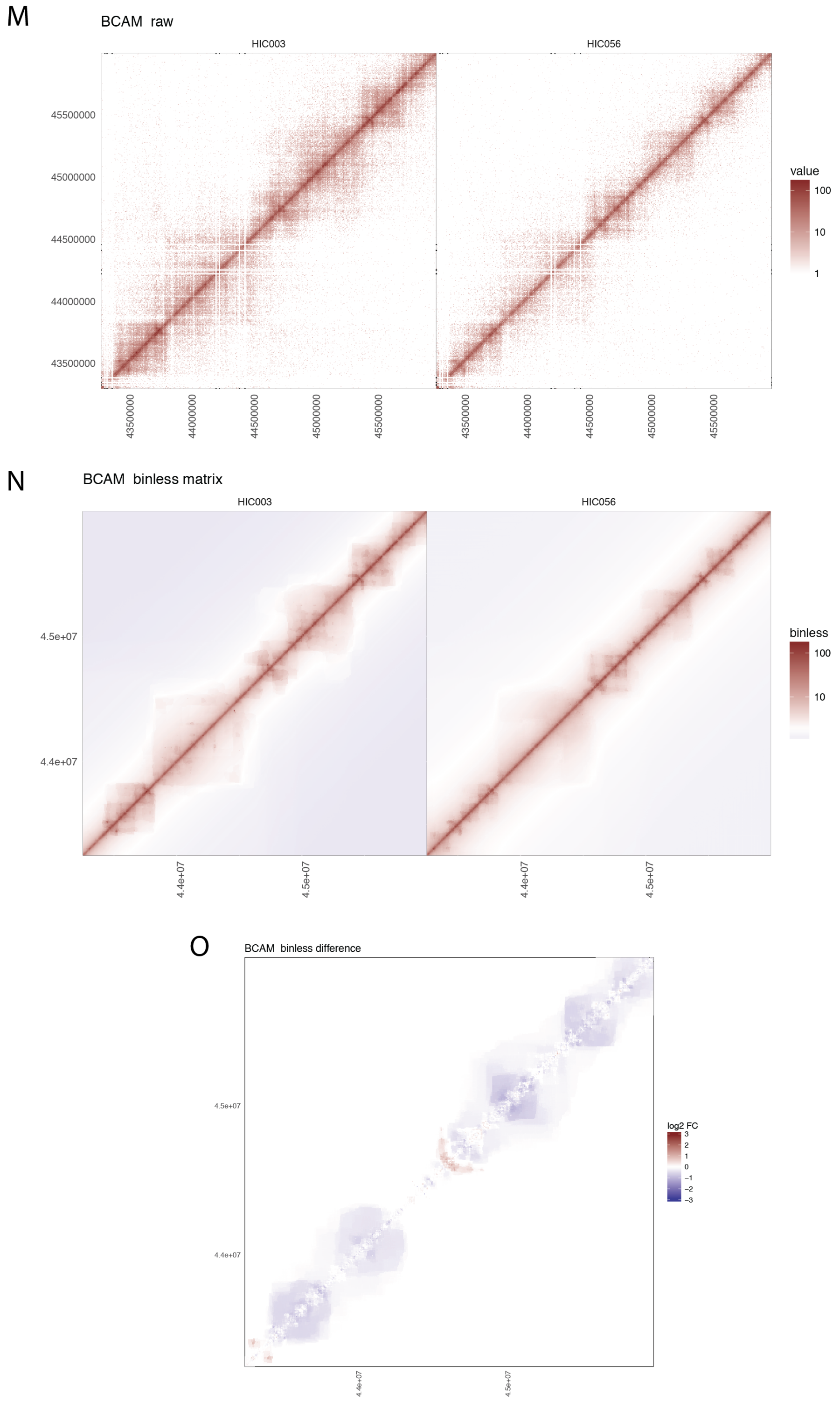

Supplementary Figure 9 (cont.). Example of difference calculations with Binless. BCAM locus (chr19:43M-46M, Rao HIC003 vs HIC056). M: Raw data. N: binless matrix. O: binless difference matrix.

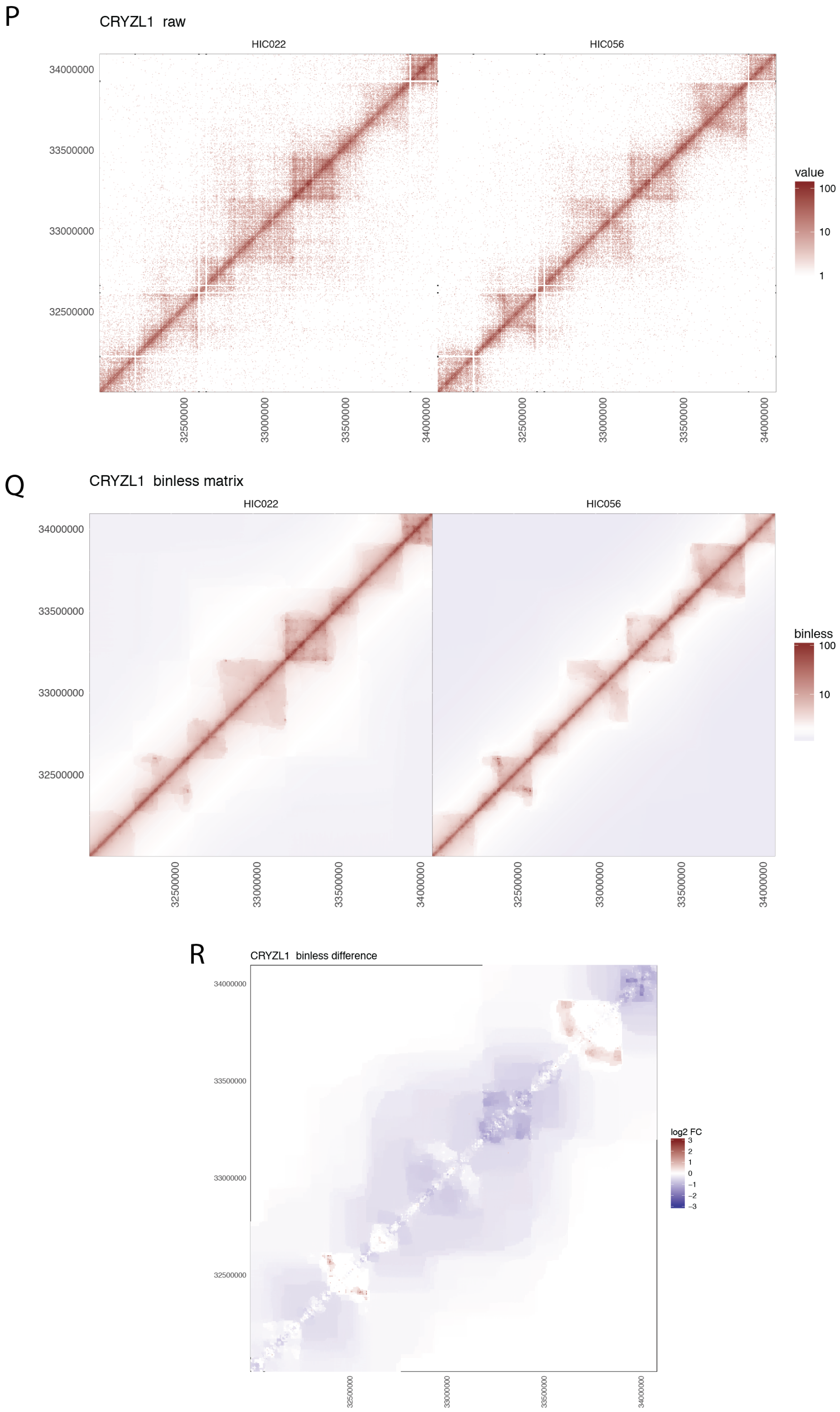

Supplementary Figure 9 (cont.). Example of difference calculations with Binless. CRYZL1 locus (chr21:32M-34M, Rao HIC022 vs HIC056). P: Raw data. Q: binless matrix. R: binless difference matrix.

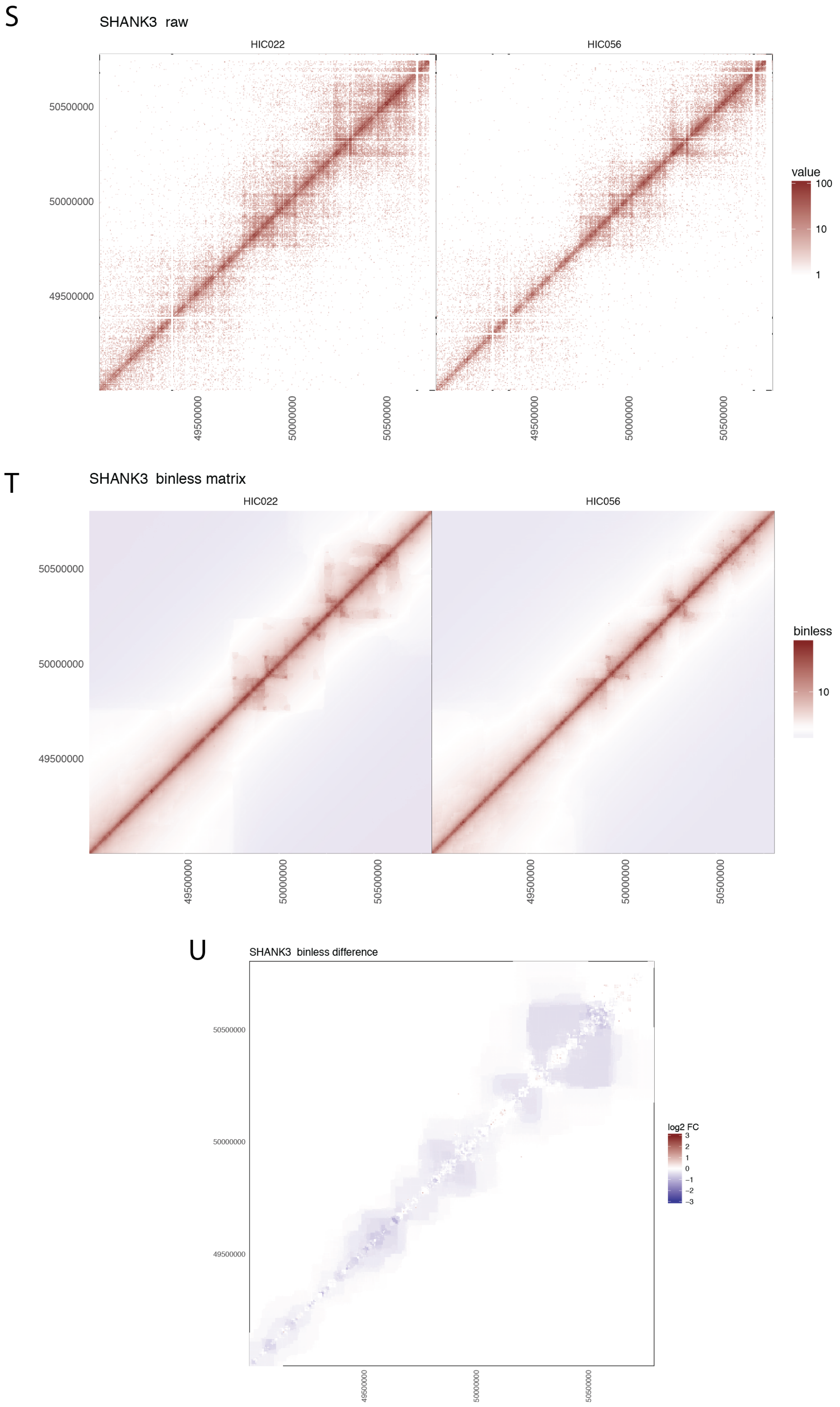

Supplementary Figure 9 (cont.). Example of difference calculations with Binless. SHANK3 locus (chr23:49M-51M, Rao HIC022 vs HIC056). S: Raw data. T: binless matrix. U: binless difference matrix.

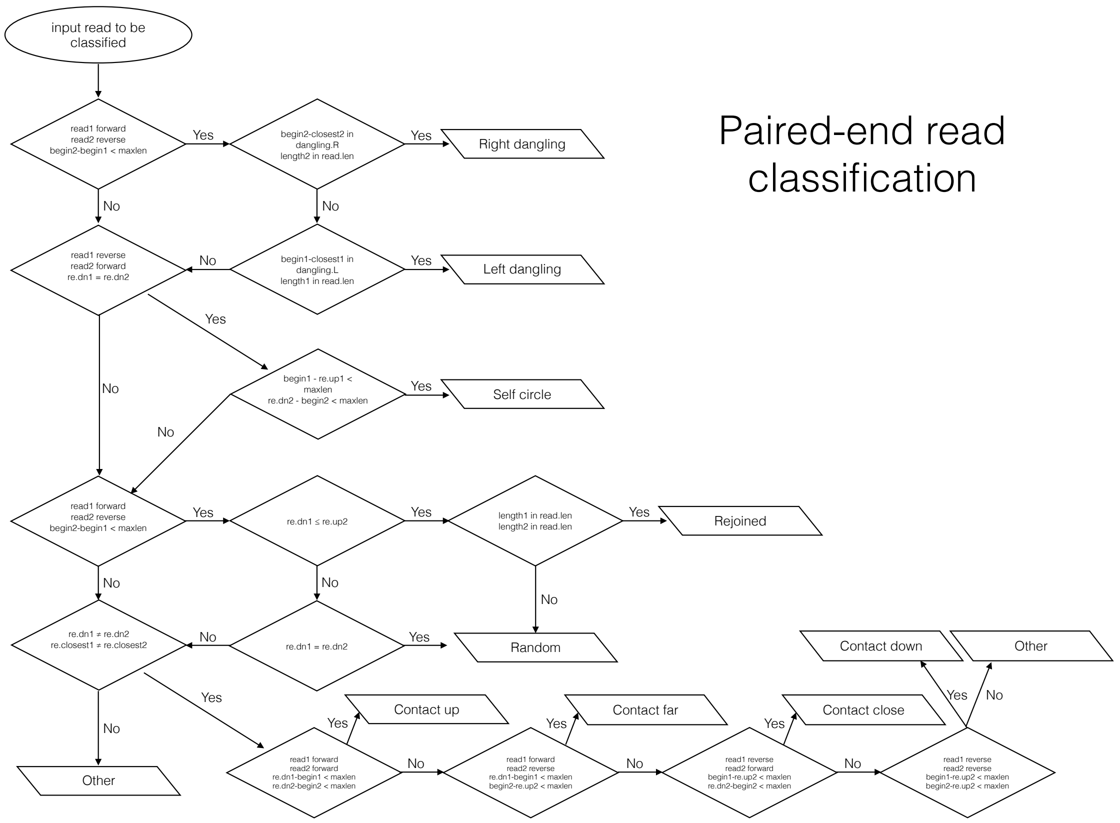

Supplementary Figure 10. Paired-end read classification. For proper classification, a number of parameters must be defined beforehand (see also Sup. Fig. 11):

- readlen: length of reads, as output by the sequencer
- maxlen: maximum size of sonication fragment
- dangling.L and dangling.R: relative positions at which restriction enzymes are expected to cut in the forward (resp. reverse) direction

The input file contains the following fields:

- begin1 and begin2: position of first base to be sequenced in read 1(resp. read 2). Here we assume begin1<begin2, but the file will be sorted to ensure this condition.
- strand information for each mapped read
- re.up1, re.up2: position of upstream restriction site
- re.dn1, re.dn2: position of downstream restriction site
- length1, length2: length of mapped sequence

And the following are generated on the fly:

- re.closest1, re.closest2: position of downstream restriction site

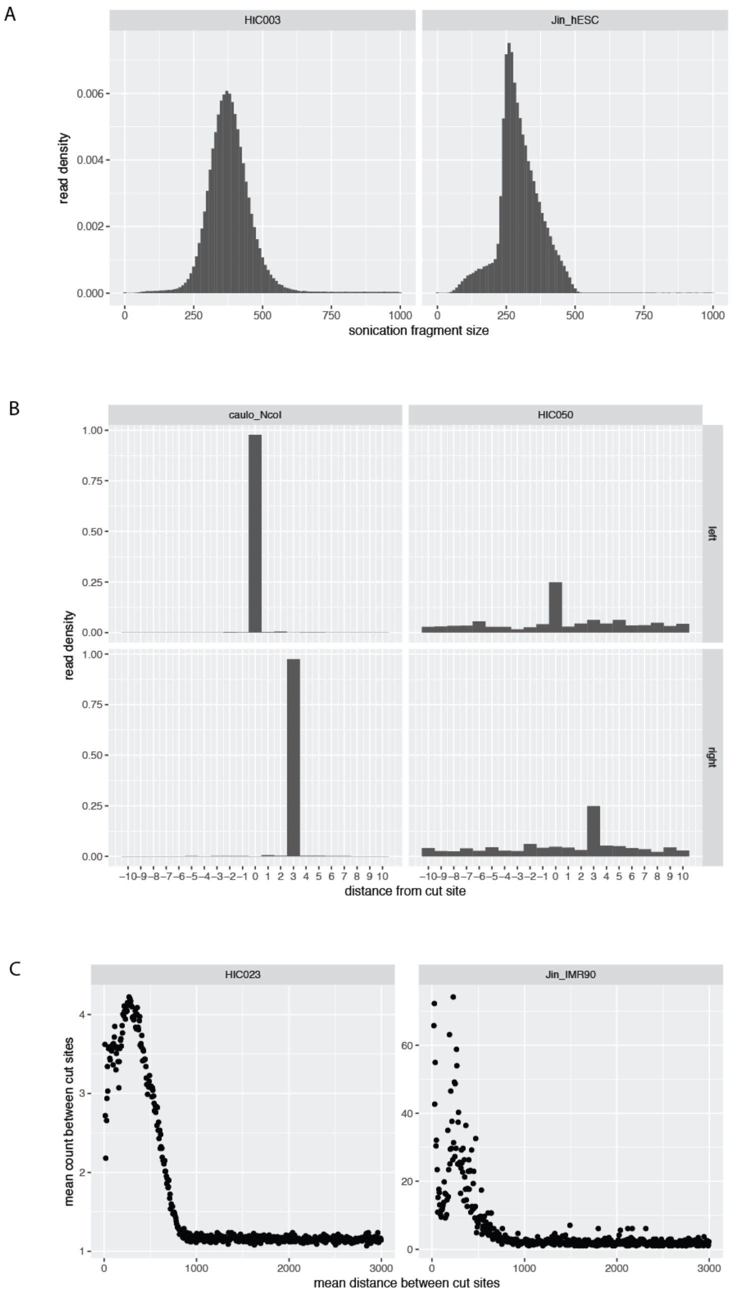

Supplementary Figure 11. Hi-C diagnostic plots. A) Distribution of the end-to-end distance of dangling, rejoined and random reads. Left: Rao HIC003 using MboI in GM12878 [4], right: Jin hESC HindIII replicate A [63]. In both experiments, the majority of sonication fragments were less than 700 base pairs in size. We can therefore set the maxlen parameter to 700 or above. B) Histogram of dangling reads around cut site. Left: Caulobacter NcoI dataset [28], right: Rao HIC050 using MboI in IMR90 [4]. Dangling ends are produced with an overhang of 4 nucleotides in both cases. Since by convention, the start of the overhang is at position 0 on the forward strand, it will be at position 3 on the reverse strand. We can therefore set the parameters dangling.L to 0 and dangling.R to 3. C) Average number of counts per cut site intersection, as a function of distance. Left: Rao HIC023 using MboI in GM12878, right: Jin IMR90 HindIII replicate F [63]. Binless does not model the increase at very small distances. We can therefore set dmin to 1000.

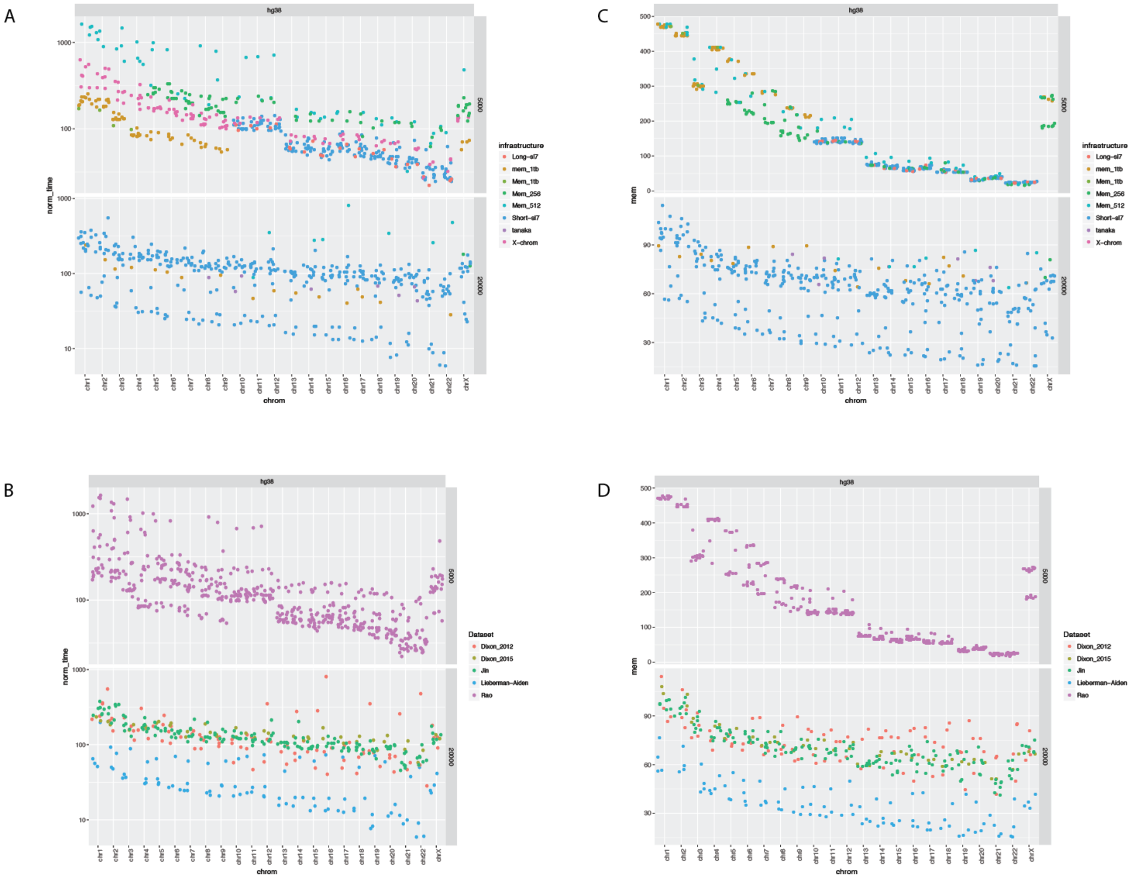

Supplementary Figure 12: Computational requirements. A: Runtime (in minutes) coloured by infrastructure. Upper panel: 5kb base resolution. Lower panel: 20kb. B: Runtime coloured by dataset. C: Memory usage (in Gb) coloured by infrastructure. D: Memory usage coloured by dataset.

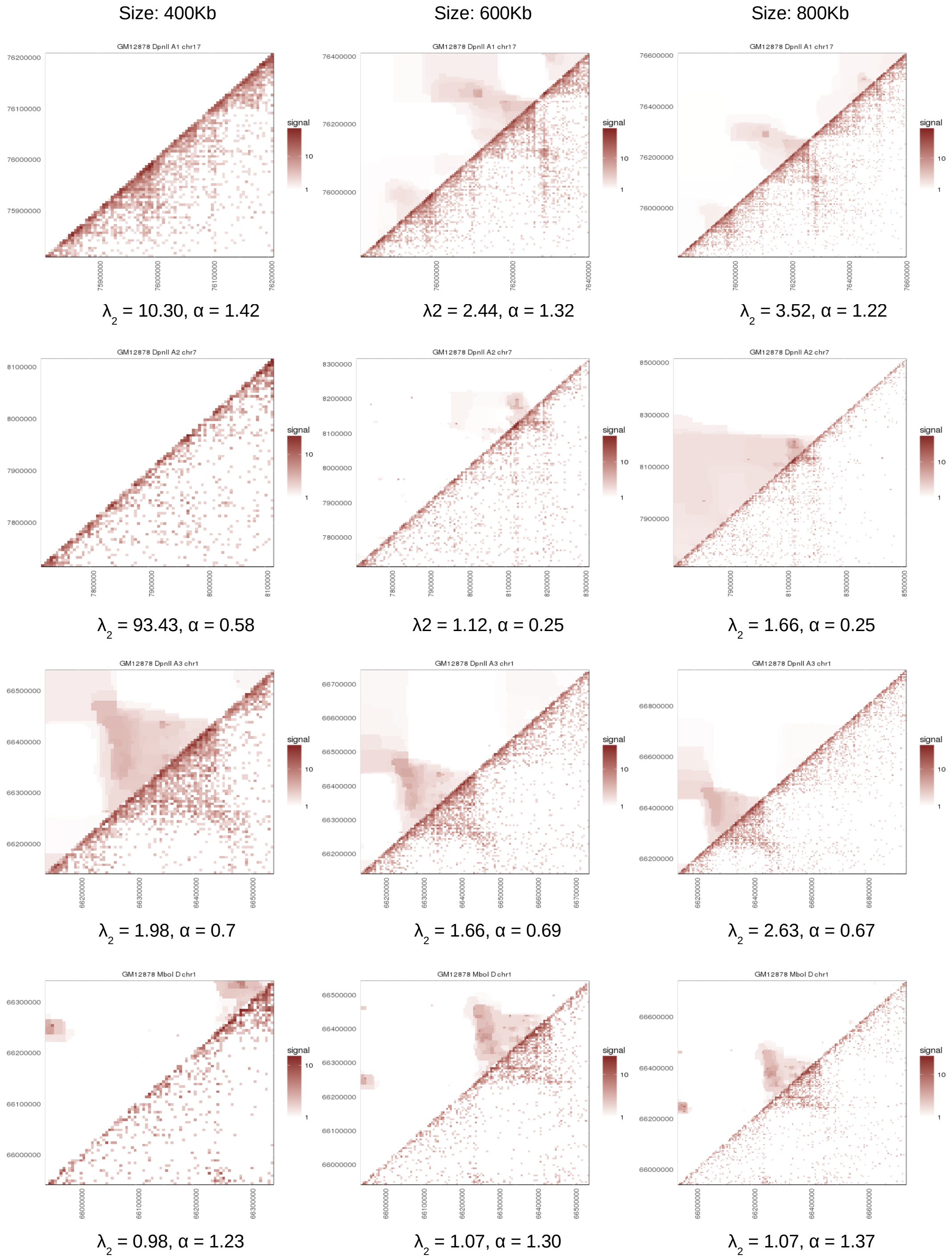

Supplementary Figure 13. Example of subset matrices to estimate parameters for fast binless. Signal detection and the corresponding values of λ_2 and α in submatrices of increasing size (columns), for successive overlapping regions (rows). Lower triangle is raw data, while upper triangle is binless signal matrix

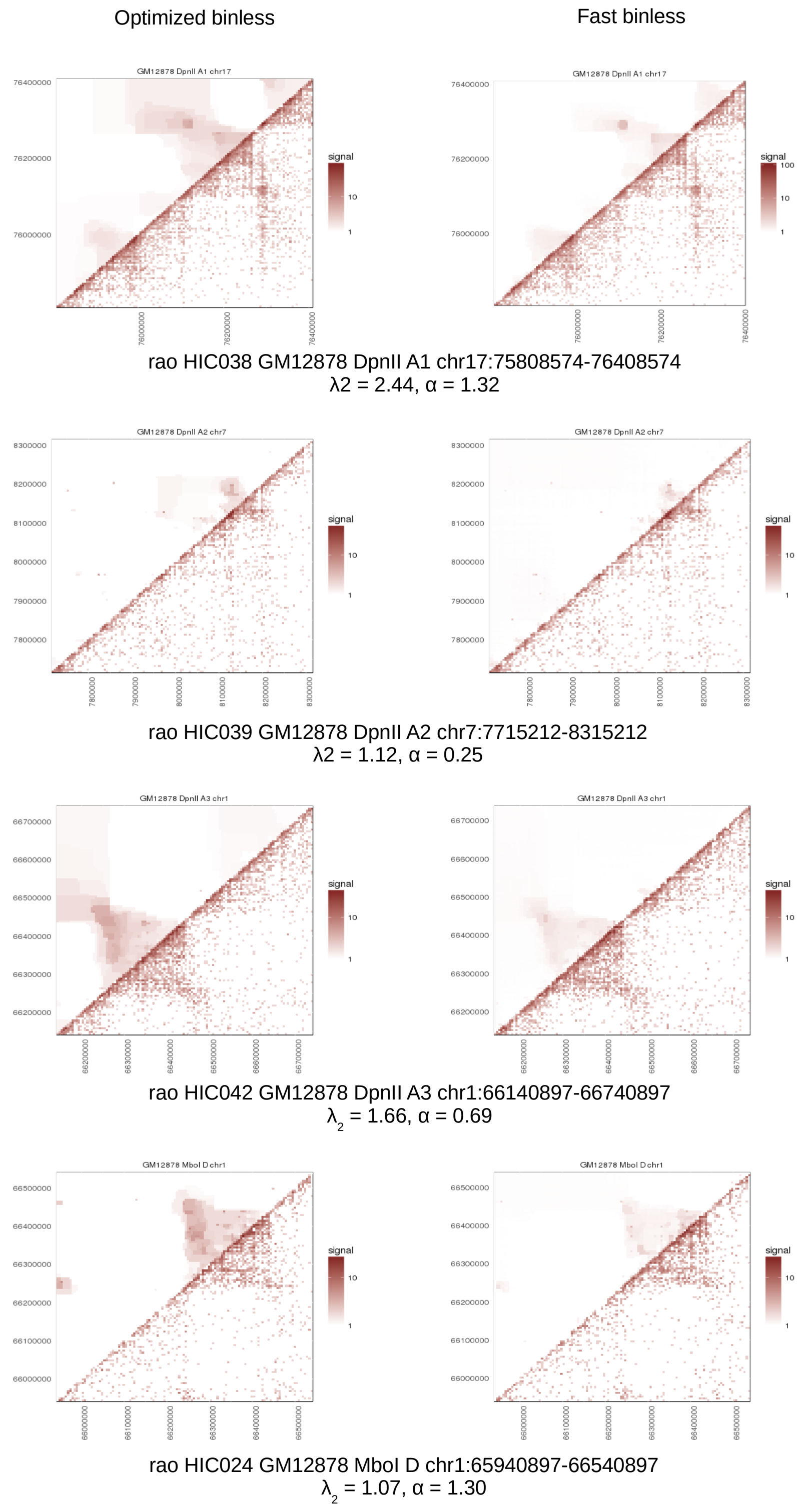

Supplementary Figure 14. Comparison of signal detection in submatrices normalized with optimized binless and the corresponding section of the full chromosome normalized with Fast binless.

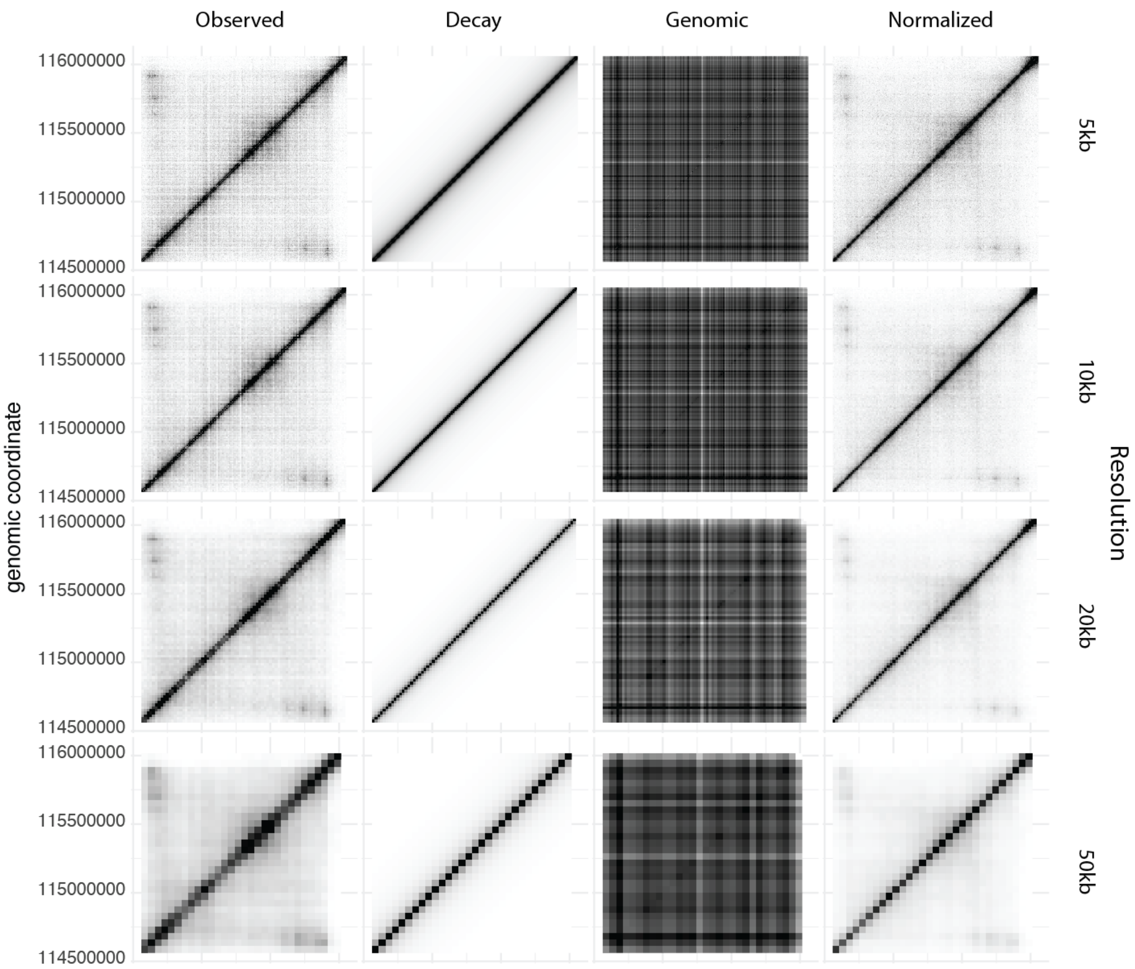

Supplementary Figure 15. Binned matrices for the TBX3 locus (chr12:114M-117M, dataset GM12878 from [4]). Although both the data and the biases are binless by definition, the matrices can be represented at different resolutions. Raw data (observed) is used to estimate the decay and the genomic biases, which are then used to normalize the final datasets to obtain the normalized matrix and its standard deviation (sd), which is an estimate of the uncertainty of the data.

Supplementary Methods

Binless normalization of Hi-C data provides significant interaction and difference detection independently of resolution

Spill *et al.*

Contents

1 Preprocessing **2**

2 Binless model **2**
2.1 Likelihoods . . . 2
2.2 Priors for the genomic and decay biases . . . 3
2.3 Prior for the signal . . . 3

3 Normalization **4**
3.1 Genomic biases . . . 5
3.2 Diagonal decay . . . 7
3.3 Exposures . . . 9
3.4 Signal . . . 9
3.5 Dispersion . . . 11
3.6 Procedure . . . 11

4 Interaction and difference detection **11**
4.1 Interaction detection . . . 11
4.2 Binless difference detection . . . 12

5 Binned matrices **13**
5.1 Available matrices . . . 13
5.2 Grouping . . . 14

6 Fast binless **14**
6.1 Model . . . 14
6.2 Estimation of parameters . . . 15

1 Preprocessing

When two cut sites are too close, the fragment that spans them can become unmappable. This lack of mappability, in turn, results in an inflation of zeros at these cut sites, a fact that is not captured by the model below. To avoid this issue, we fuse cut sites that are closer than a minimum distance (dfuse, default 5 bases). The position of the new cut site is taken as the mean of the position, rounded to the nearest integer. Observed counts are taken to be the sums of the respective counts of each cut site intersection.

PCR artifacts can result in strong long-distance interactions that have no relevance to what should be detected. To remove these artifacts, we fit the histogram built by aggregating count*log(distance) in 20 bins of equal size, where count is the number of reads in one of the four count categories, at each cut site intersection. From largest to lowest count*log(distance), we mark all counts as artifacts as long as they all fall above the fitted line. The value of the count is then set to 1 for these artifacts.

2 Binless model

2.1 Likelihoods

Let DL_i be the number of left-dangling reads at cut site i. Similarly, write DR_i and RJ_i for right-dangling and rejoined reads. Let c_{ij}^{far} be the number of reads found in the "contact far" category (Fig. 6D) between cut sites i and j. Similarly, define c_{ij}^{close} for "contact close", c_{ij}^{up} for "contact up" and c_{ij}^{down} for "contact down. The exact model takes the form of the following negative binomial regression

$$RJ_i \sim \text{NB}\left(e^{e_{RJ}}\sqrt{\rho_i \iota_i}, \alpha\right) \qquad DL_i \sim \text{NB}\left(e^{e_{DE}}\iota_i, \alpha\right) \qquad DR_i \sim \text{NB}\left(e^{e_{DE}}\rho_i, \alpha\right) \tag{1}$$

$$\begin{aligned} c_{ij}^{\text{far}} &\sim \text{NB}\left(e^{e_C}\iota_i\rho_j f_{ij} s_{ij}, \alpha\right) \qquad & c_{ij}^{\text{down}} &\sim \text{NB}\left(e^{e_C}\rho_i\rho_j f_{ij} s_{ij}, \alpha\right) \\ c_{ij}^{\text{up}} &\sim \text{NB}\left(e^{e_C}\iota_i\iota_j f_{ij} s_{ij}, \alpha\right) \qquad & c_{ij}^{\text{close}} &\sim \text{NB}\left(e^{e_C}\rho_i\iota_j f_{ij} s_{ij}, \alpha\right) \end{aligned} \tag{2}$$

where i and j are two cut sites with $i < j$. In our parametrization, $\text{NB}(x, \alpha)$ has mean x and variance $x + x^2/\alpha$. In this Generalized Additive Model (GAM) [1–3], $\log \iota$, and $\log \rho$ are smooth functions of the genomic position, $\log f$ is a smooth monotonically decreasing function of the log distance between cut sites, in bases, and $\log s$ is a smooth and sparse signal estimate. Counts, dangling ends and rejoined ends are given a different offset (e^{e_C}, $e^{e_{DE}}$, $e^{e_{RJ}}$). Each dataset is given a separate offset, to account for differences in sequencing depth. Datasets with different restriction enzymes have different sets of genomic biases (ι and ρ). Depending on context, they can also be given a different diagonal decay (f), a choice left to the user. If differences in the diagonal decay are relevant to the analysis, only one decay should be modelled; if differences are assumed to have a purely technical origin (such as using

two restriction enzymes with very different cutting paterns), each dataset should be assigned a different decay. Note that to avoid modelling too small distances, a minimum distance is introduced, and all contacts below that threshold (dmin, default 1kb) are not modelled. In addition, we fuse cut sites that are closer than a given distance (default 5 bases). In the remaining of this section, we assume to simplify the notation that there is only one dataset.

2.2 Priors for the genomic and decay biases

To obtain the desired smoothness of $\log \iota$, $\log \rho$ and $\log f$, we we follow the Bayesian formulation of GAM by Lang and Bretzger [4, 5] with some modifications. Each smooth is built with a cubic spline base on the independent variable, as is done traditionally in the P-spline formulation [6] of GAM [2]. Let g be one such smooth built on the variables $\{x_i\}_{1 \leqslant i \leqslant N}$, and $\mathbf{g} \equiv (g_1, \dots, g_N)$ with $g_i \equiv g(x_i)$. In the cubic spline base, $g_i = \sum_{j=1}^{K} \beta_j B_j(x_i)$ where B_j is one of K cubic splines placed at equidistant knots [6]. Equivalently, we can write $\mathbf{g} \equiv \mathbf{X_g} \boldsymbol{\beta}_\mathbf{g}$, where $\mathbf{X_g}$ is a $N \times K$ matrix and $\boldsymbol{\beta}_\mathbf{g}$ the coefficients on the spline. For the genomic splines, we place a standard normal distribution on $\boldsymbol{\beta}_\mathbf{g}$. For all splines, we place a degenerate normal distribution on the second-order differences on $\boldsymbol{\beta}_\mathbf{g}$ with zero mean and variance $1/(K\lambda_g)$. Finally, we place a half-normal hyperprior on λ_g, as recommended for variance parameters [7], with $\sigma = 1$ for the decay spline, and $\sigma = 10^{-4}$ for the genomic splines.

The prior on the differences on $\boldsymbol{\beta}_\mathbf{g}$ amounts to a uniform prior on two coefficients, and a well-defined normal distribution on the remaining coefficients [2, section 4.8.2 p. 187]. The scaling factor K is there to ensure that the values of the penalties are approximately independent of the number of basis functions and the span of the spline.

Identifiability is ensured by enforcing the mean of each smooth to be zero. We choose not to incorporate this nor any other constraint in the design matrix, but instead push it to the coefficients. For the genomic biases ι and ρ, this allows us to exploit the sparsity of $\mathbf{X}$, owing to the compact support of cubic splines. We use a default of 50 basis function per kilobase. For the monotonically decreasing spline f, we constrain the parameters $\boldsymbol{\beta}_\mathbf{f}$ to be ordered. We use a default of 10 basis functions per distance decade.

2.3 Prior for the signal

Signal modelling is made with the help of the gfl package [8], which is a fast and parallelizable implementation of the weighted fused lasso on a graph. We bin the data at a fixed resolution and place the lasso coefficients on a triangle grid (the Hi-C matrix is symmetric), penalizing neighboring coefficients (penalty λ_2). Sparsity of the solution is enforced by placing an L1 prior on the coefficients themselves (penalty λ_1). It can be computed for any $\lambda_1 > 0$ by soft-thresholding the solution for $\lambda_1 = 0$, owing to the fact that grid cells do not overlap.

Since we are not aware of a proper prior for the 2D fused lasso on a triangle grid, we use the Bayesian Information Criterion (BIC) to optimize the fusion, sparsity and offset parameters. The sparsity penalty λ_1 cannot be smaller than the target precision. The fusion penalty λ_2 cannot be smaller than 0.1. Because we want to represent the excess signal compared to the local background, we impose $\log s \geqslant 0$, which is an additional constraint on λ_1 and the offset.

3 Normalization

For N cut sites, there are $2N(N-1)+3N$ likelihoods to be evaluated. To avoid this quadratic scaling, we use the Iteratively Re-weighted Least Squares (IRLS) approximation. The IRLS allows to compute an exact solution to regression problems based on distributions from the exponential family [2, pp. 63-67]. A single evaluation of a costy likelihood is replaced by a quick iteration of least squares estimates followed by weight updates. Using the property that products of normal likelihoods are themselves normal, we reduce the number of likelihoods to 5N in the IRLS cycle, as we now explain.

We start with the normal approximation to the log likelihood of the counts (eq. 2), as is done for distributions from the exponential family [2, pp. 63-67]. Write $c_{ij} \sim \mathrm{NB}(\mu_{ij}, \alpha)$. Then the IRLS approximation states that, at iteration k, we can optimize the objective function

$$S^{[k]} = -\frac{1}{2}\sum_{i=1}^{N-1}\sum_{j=i+1}^{N} \frac{(z_{ij}^{[k]} + \log \mu_{ij}^{[k]} - \log \mu_{ij})^2}{{\sigma_{ij}^{[k]}}^2} \tag{3}$$

where the superscript [k] indicates that the coresponding parameters are held fixed at the initial value at iteration k, and

$$z_{ij}^{[k]} \equiv \frac{c_{ij} - \mu_{ij}^{[k]}}{\mu_{ij}^{[k]}} \tag{4}$$

$$\sigma_{ij}^2 \equiv \frac{1}{\mu_{ij}^2}\left(\mu_{ij} + \frac{\mu_{ij}^2}{\alpha}\right) \tag{5}$$

$S^{[k]}$ has the same first and second derivatives with respect to $\log \mu_{ij}$ as the log likelihood (eq. 2) at the maximum. In other words, if the dispersion is held constant, a likelihood of the form $c_{ij} \sim \mathrm{NB}(\mu_{ij}, \alpha)$ can be replaced by $z_{ij}^{[k]} + \log \mu_{ij}^{[k]} \sim \mathcal{N}(\log \mu_{ij}, \sigma_{ij}^{[k]})$.

3.1 Genomic biases

IRLS likelihood Write $\mu_{ij} = e^{e_C} a_i b_{ij}$. We hold all parameters relative to index j fixed during iteration k. Therefore $S^{[k]}$ simplifies as

$$S_g^{[k]} = -\frac{1}{2} \sum_{i=1}^{N} \sum_{\substack{j=1 \\ j \neq i}}^{N} \frac{(z_{ij}^{[k]} + \eta_i^{[k]} - \eta_i)^2}{2\sigma_{ij}^{[k]2}} \tag{6}$$

$$\eta_i \equiv e_C + \log a_i \tag{7}$$

Grouping all terms involving j, we get

$$S_g^{[k]} = -\frac{1}{2} \sum_{i=1}^{N} \left[\sum_{\substack{j=1 \\ j \neq i}}^{N} \frac{(z_{ij}^{[k]} + \eta_i^{[k]} - \hat{\eta}_i^{[k]})^2}{2\sigma_{ij}^{[k]2}} + \frac{(\hat{\eta}_i^{[k]} - \eta_i)^2}{\sigma_i^{[k]2}} \right] \tag{8}$$

$$\hat{\eta}_i^{[k]} \equiv \frac{\sum_{\substack{j=1 \\ j \neq i}}^{N} (z_{ij}^{[k]} + \eta_i^{[k]})/\sigma_{ij}^{[k]2}}{\sum_{\substack{j=1 \\ j \neq i}}^{N} 1/\sigma_{ij}^{[k]2}} \qquad \frac{1}{\sigma_i^{[k]2}} \equiv \frac{1}{2} \sum_{\substack{j=1 \\ j \neq i}}^{N} \frac{1}{\sigma_{ij}^{[k]2}} \tag{9}$$

Therefore, to optimize the genomic biases, we can use the approximate genomic likelihood

$$\hat{\eta}_i^{[k]} \sim \mathcal{N}\left(\eta_i, \sigma_i^{[k]}\right) \tag{10}$$

However, since the model likelihoods involve either ι or ρ, we write

$$\hat{\eta}L_i^{[k]} \sim \mathcal{N}\left(\eta L_i, \sigma L_i^{[k]}\right) \qquad \hat{\eta}R_i^{[k]} \sim \mathcal{N}\left(\eta R_i, \sigma R_i^{[k]}\right) \tag{11}$$

$$\eta L_i \equiv e_C + \log \iota_i \qquad \eta R_i \equiv e_C + \log \rho_i \tag{12}$$

The definitions of $\hat{\eta}L_i^{[k]}$ (resp. $\hat{\eta}R_i^{[k]}$) and $\sigma L_i^{[k]}$ (resp. $\sigma R_i^{[k]}$) is identical to previously, except that the sums run over "far" and "up" counts (resp. "close" and "down") when $j > i$ and over "close" and "up" counts (resp. "far" and "down") when $j < i$.

For rejoined and dangling ends, we define

$$\mu RJ_i \equiv e^{e_{RJ}} \sqrt{\iota_i \rho_i} \quad \mu DL_i \equiv e^{e_{DE}} \iota_i \quad \mu DR_i \equiv e^{e_{DE}} \rho_i \tag{13}$$

and, with T in place of RJ, DL or DR, we define the IRLS likelihoods

$$\hat{\eta}T_i^{[k]} \sim \mathcal{N}\left(\eta T_i, \sigma T_i^{[k]}\right) \tag{14}$$

$$\eta T_i \equiv \log \mu T_i \tag{15}$$

$$\hat{\eta}T_i \equiv \frac{T_i}{e^{\eta T_i}} - 1 + \eta T_i \tag{16}$$

$$\sigma T_i^2 \equiv \frac{1}{\mu T_i} + \frac{1}{\alpha} \tag{17}$$

To speed up the calculation, we take advantage that the vast majority of the counts c_{ij} are usually zero. We do not store these counts explicitly. Instead, we track the number of zeros per distance bin, signal bin and cut site side (left or right). We use $z_{ij} = -1$ and $\mu_{ij} = \exp(e_C) b_i f_l s_k$, where b_i is the bias (ι or ρ) of cut site i, f_l is the decay at distance bin l and s_k the signal contribution at signal bin k (see below).

Posterior For the genomic biases, there is only a centering constraint. There is no offset, since it is fitted separately. Further, the posterior is separable in datasets which have different enzymes, since they will have different biases. We therefore compute their IRLS updates separately. For datasets having a common bias, the previous section showed the data can be put in a vector $\hat{\eta}T^{[k]}$ of size N (assuming N distinct cut sites across these datasets). There are two biases to model, ι and ρ, which should be centered with respect to the weight vector $w \equiv 1_N$. Dropping the subscript k, define

$$y \equiv \begin{pmatrix} \hat{\eta}RJ \\ \hat{\eta}DL \\ \hat{\eta}DR \\ \hat{\eta}L \\ \hat{\eta}R \end{pmatrix} \qquad W \equiv \begin{pmatrix} 0 & 0 \\ \vdots & \vdots \\ 0 & 0 \\ 1 & 0 \\ \vdots & \vdots \\ 1 & 0 \\ 0 & 1 \\ \vdots & \vdots \\ 0 & 1 \end{pmatrix} \tag{18}$$

where y is a vector of size 5N and W is a $5N \times 2$ matrix with the first 3N rows equal to zero. Assume we can write

$$X \equiv \begin{pmatrix} X_\iota & X_\rho \end{pmatrix} \equiv \begin{pmatrix} X_1/2 & X_1/2 \\ X_1 & 0 \\ 0 & X_1 \\ X_1 & 0 \\ 0 & X_1 \end{pmatrix} \qquad D \equiv \begin{pmatrix} \lambda_\iota D_1 & 0 \\ 0 & \lambda_\rho D_1 \end{pmatrix} \qquad \beta \equiv \begin{pmatrix} \beta_\iota \\ \beta_\rho \end{pmatrix} \tag{19}$$

with X_1 being a $N \times K$ cubic spline base (X_{1ij} is the cubic spline j evaluated at the position of cut site i), X $5ND \times 2K$, D_1 a $(K-2) \times K$ second-order difference matrix, D $(2K-4) \times 2K$ and β a vector of size $2K$. The two centering constraints are $1^\top X_\iota \beta_\iota = 0$ and $1^\top X_\rho \beta_\rho = 0$, or equivalently $W^\top X\beta = 0$ where W selects either of the two biases. Then, incorporating the constraint with the Lagrange multiplier $\mu \equiv (\mu_\iota, \mu_\rho)^\top$, the log posterior is

$$\mathcal{L} = -\frac{1}{2}(y - X\beta)^\top S^{-2}(y - X\beta) - \mu^\top W^\top X\beta + \sum_{i=\iota,\rho}\left[-\frac{K^2\lambda_i^2}{2}\beta_i^\top D_1^\top D_1 \beta_i + (K-2)\log\lambda_i - \frac{\lambda_i^2}{2\sigma^2}\right] \tag{20}$$

We solve for μ, β and λ, yielding

$$A \equiv X^\top S^{-2} X + K^2 D^\top D \tag{21}$$

$$\Gamma \equiv \mathbb{1} - X^\top W (W^\top X A^{-1} X^\top W)^{-1} W^\top X A^{-1} \tag{22}$$

$$\beta \equiv A^{-1}\Gamma X^\top S^{-2} y \tag{23}$$

$$\lambda_i = \sqrt{\frac{K-2}{K^2\beta_i^\top D_1^\top D_1\beta_i + 1/\sigma^2}} \quad i = \iota, \rho \tag{24}$$

Implementation notes X_1 is band diagonal and therefore X is sparse. We perform a simplicial Cholesky decomposition of A. We follow the performance iteration scheme [2], in which we update λ_i at each IRLS step. Final estimates of $\log\iota = X_1\beta_\iota$ and $\log\rho = X_1\beta_\rho$ are centered after a complete IRLS estimation, because we observe it gives better numerical stability.

3.2 Diagonal decay

IRLS likelihood To estimate the diagonal decay, we proceed similarly to previously, with the difference that we bin the log-distance to fractions of the support of each basis function (by default, we use 10 bins per basis function). Assume that f_{ij} is constant equal to f_l within such a bin l, with B_l being the set of corresponding indices. The distance of this bin is taken to be the geometric mean of the distance of all counts falling in this bin. An otherwise identical procedure leads to the approximate likelihood

$$\hat{\kappa}_l^{[k]} \sim \mathcal{N}\left(\kappa_l, \sigma_l^{[k]}\right) \tag{25}$$

$$\kappa_l \equiv e_C + \log f_l \tag{26}$$

$$\hat{\kappa}_l^{[k]} \equiv \frac{\sum_{(i,j)\in B_l}(z_{ij}^{[k]} + \kappa_l^{[k]})/\sigma_{ij}^{[k]2}}{\sum_{(i,j)\in B_l} 1/\sigma_{ij}^{[k]2}} \tag{27}$$

$$\frac{1}{\sigma_l^{[k]2}} \equiv \sum_{(i,j)\in B_l} \frac{1}{\sigma_{ij}^{[k]2}} \tag{28}$$

where the sums are understood to span over all four count types. Note that to match the original definition of e_C, we have $\sum_l |B_l| \log f_l = 0$ where $|B_l|$ is the number of cut-sites in distance bin B_l. Again, we take advantage that the vast majority of the counts c_{ij} are usually zero to compute $\hat{\kappa}_i$ efficiently.

Posterior The decay is centered and constrained to decrease. We use quadratic programming to enforce the monotonicity, but leave the centering to a final adjustment. The centering matrix is

$$C \equiv \begin{pmatrix} 1 & 0 & \cdots & 0 \\ -1 & 1 & \ddots & \vdots \\ 0 & -1 & \ddots & 0 \\ \vdots & \ddots & \ddots & 1 \\ 0 & \cdots & 0 & -1 \end{pmatrix} \tag{29}$$

C is $K \times (K-1)$ and the constraint is $C^\top \beta \geqslant 0$. The multipliers are $\Lambda^\top \equiv (\lambda_1, \cdots, \lambda_{K-1})$ for C. The extended target is

$$\begin{aligned} \mathcal{L} = &-\frac{1}{2}(\hat{\kappa} - X\beta)^\top S^{-2}(\hat{\kappa} - X\beta) \\ &- \frac{K^2\lambda^2}{2}\beta^\top D^\top D\beta + (K-2)\log\lambda - \frac{\lambda^2}{2\sigma^2} - \Lambda^\top C^\top \beta \end{aligned} \tag{30}$$

where X is a $N \times K$ cubic spline base (assuming there are N decay bins and K basis functions) and D is a $(K-2) \times K$ second-order difference matrix. The solution for λ can be given explicitly

$$\lambda = \sqrt{\frac{K-2}{K^2\beta^\top D^\top D\beta + 1/\sigma^2}} \tag{31}$$

Implementation notes We use the quadprog R package to compute the solution to β. We follow the performance iteration scheme [2], in which we update λ_i at each IRLS step. The final estimate of $\log f = X\beta$ is centered after a complete IRLS estimation. For the centering weights, we use the number of contacts in each decay bin.

3.3 Exposures

To estimate the exposures e_C, e_{RJ} and e_{DE} for each dataset, we proceed similarly to previously. Here the problem reduces to one group per count type and dataset. Since there is only a uniform prior on each coefficient, we can give the final estimates with the IRLS likelihood alone

$$e_{d,T}^{[k+1]} \equiv \frac{\sum_{i \in D_{d,T}} (z_i^{[k]} + e_{d,T}^{[k]}) / \sigma_i^{[k]2}}{\sum_{i \in D_{d,T}} 1/\sigma_i^{[k]2}} \tag{32}$$

where $D_{d,T}$ is the collection of indices pertaining to dataset d and count type $T = RJ, DE, C$.

3.4 Signal

IRLS likelihood In this step, we update both the signal and the count offsets e_C. We bin the genome in regularly spaced intervals (default 5k base resolution). Therefore, every cut-site intersection falls into a pair of bins. The signal s is assumed to be constant in such a pixel, with $B_{ll'}$ being the set of corresponding cut-site indices. An otherwise identical procedure leads to the approximate likelihood

$$\hat{\phi}_{ll'}^{[k]} \sim \mathcal{N}\left(\phi, \sigma_{ll'}^{[k]}\right) \tag{33}$$

$$\phi_{ll'} \equiv \log s_{ll'} \tag{34}$$

$$\hat{\phi}_{ll'}^{[k]} \equiv \frac{\sum_{(i,j) \in B_{ll'}} (z_{ij}^{[k]} + \phi_{ll'}^{[k]}) / \sigma_{ij}^{[k]2}}{\sum_{(i,j) \in B_{ll'}} 1/\sigma_{ij}^{[k]2}} \tag{35}$$

$$\frac{1}{\sigma_{ll'}^{[k]2}} \equiv \sum_{(i,j) \in B_{ll'}} \frac{1}{\sigma_{ij}^{[k]2}} \tag{36}$$

where the sums are understood to span over all four count types. Again, we take advantage that the vast majority of the counts c_{ij} are usually zero to compute $\hat{\phi}_{ll'}$ efficiently.

Because some portions of the data are not captured well by the background model, we set their contribution during signal estimation to zero. For that purpose, we form

a genomic z-score Z_l by standardizing the following quantity

$$\tilde{Z}_l \equiv \frac{\sum_{(i,j)\in R_l} z_{ij}/\sigma_{ij}}{\sum_{(i,j)\in R_l} 1/\sigma_{ij}^2} \tag{37}$$

Here, $R_l \equiv \bigcup_{l'} B_{ll'}$. We set $\sigma_{ll'}^{[k]} = +\infty$ for all signal bins for which the two-tailed probablility of observing Z_l or a more extreme value is lower than q_{min} (0.01 by default). In addition, all signal bins that contain data below the minimum distance (dmin, default 1kb) are discarded as well.

Posterior Writing $b = ll'$ and dropping the superscript k, the target is

$$\mathcal{L} = -\frac{1}{2}\sum_b \left(\frac{\hat{\phi}_b - \phi_b - e'_C}{\sigma_{s_b}}\right)^2 - \lambda_2 \sum_{b,b' \text{ neighbors}} |\phi_b - \phi_{b'}| - \sum_b \lambda_1 |\phi_b| \tag{38}$$

During normalization, we maintain $\lambda_2 = 2.5$, and estimate ϕ_b, e'_C and λ_1. We only perform one IRLS iteration. In absence of a proper prior for the 2D weighted graph fused lasso, we use the BIC to optimize λ_1 and e'_C

$$\text{BIC} = \frac{1}{2}\sum_b \left(\frac{\hat{\phi}_b - \tilde{\phi}_b(\lambda_1) - e'_C}{\sigma_{s_b}}\right)^2 + \log(n_{obs})\text{dof}(\lambda_1) \tag{39}$$

with $\tilde{\phi}_b(\lambda_1) = S_{\lambda_1}(\phi_b)$ the solution of the fused lasso soft-thresholded at λ_1, n_{obs} the total number of cut site intersections considered, and $\text{dof}(\lambda_1)$ the degrees of freedom of the fused lasso, i.e. the total number of nonzero patches of constant $\tilde{\phi}_b$. Finally, we update e_C by adding e'_C to the previous estimate.

Implementation We use the gfl package [8] to compute the solution for ϕ at $\lambda_1 = e'_C = 0$ and λ_2 fixed. For the tolerance, we use a value 20 times stricter than the one requested for the general computation (default 0.005). Then, we estimate λ_1 and e'_C simultaneously as follows. We first build a sorted list of n independent values for ϕ_b, augmented by a larger element: $\beta_1 < \beta_2 < \ldots < \beta_n < \beta_{n+1} \equiv \beta_n + 1$. Then, for each pair of successive values, we set

$$\lambda_1 = \frac{\beta_{i+1} - \beta_i}{2} \qquad e'_C = \frac{\beta_{i+1} + \beta_i}{2} \tag{40}$$

and compute the BIC. We retain the values for λ_1 and e'_C which have the smallest BIC.

Computation of the degrees of freedom is performed as follows. We first build a graph with as many vertices as there are bin pairs (i.e. the dimension of ϕ). We connect two vertices b and b' both if they are neighbors, and if $|\phi_b - \phi'_b|$ is smaller than the general tolerance. The degrees of freedom is the number of connected

components of the graph.

3.5 Dispersion

The IRLS applies only to the exponential family, to which the negative binomial belongs at fixed dispersion. In this step therefore, we estimate the dispersion α by a maximum likelihood procedure on a subset of the counts. All counts and biases are taken from a number of rows (default 100) and their estimated means are computed. For each row, a maximum likelihood estimate of the dispersion is computed using a procedure adapted from the MASS R package. The retained dispersion is the median of these estimates. This estimate cannot take values smaller than a threshold (0.01 by default).

3.6 Procedure

Sup. Fig. 1b presents a flowchart of the normalization procedure. The estimation of all parameters is achieved by optimizing the diagonal decay, the genomic biases, the signal and the remaining parameters in an iterative way. To initialize the estimation, we fix the dispersion (default 0.1), the values of the exposures, ι, ρ and f to their Poisson estimates, and $\log s$ is set to zero. First, the background model is fitted while the signal component is held zero. For speed purposes, the residuals (z_{ij} and σ_{ij} variables above) are computed only once per iteration. Once converged (or reached a maximum of 5 steps by default), the decay is held constant and the signal is fitted concurrently with the unconstrained genomic biases, the exposures and the dispersion. Residuals are computed twice, once for background estimates, and once for the signal. The algorithm is run until the specified number of coordinate descent iterations is reached or until all biases change less than a relative threshold (default 10^{-1}). It is advised to check that convergence has been achieved, at least by verifying that the four objectives of the four separate optimizations reach a plateau.

4 Interaction and difference detection

4.1 Interaction detection

Interaction detection is similar to the procedure performed during normalization, but only seeks to optimize the signal ϕ and corresponding parameters λ_1 and λ_2. All other parameters are held fixed. Using the same conventions as in section 3.4, we write the target as

$$\mathcal{L} = -\frac{1}{2}\sum_b \left(\frac{\hat{\phi}_b - \phi_b}{\sigma_{s_b}}\right)^2 - \lambda_2 \sum_{b,b' \text{ neighbors}} |\phi_b - \phi_{b'}| - \sum_b \lambda_1 |\phi_b| \tag{41}$$

with the constraint that $\phi_b \geqslant 0$. During interaction detection, we optimize λ_2 and λ_1 at each IRLS iteration. Again, we use the BIC to optimize λ_2 and λ_1

$$\mathrm{BIC} = \frac{1}{2}\sum_b \left(\frac{\hat{\phi}_b - \tilde{\phi}_b(\lambda_1)}{\sigma_{s_b}}\right)^2 + \log(n_{\mathrm{obs}})\mathrm{dof}(\lambda_1) \tag{42}$$

λ_2 is optimized while holding λ_1 fixed and equal to zero. First, a rough minimum of the BIC is found by gridding. 25 log-spaced values for λ_2 are generated between 0.1 and 100. The values flanking the minimum are used as boundaries for the refinement. We use the one-dimensional optimization function provided by R for that purpose. By default, 50 IRLS iterations are performed at most for every evaluation at a given λ_2.

Optimization of λ_1 is identical to what has been described in section 3.4, with the exception that the minimum admissible value of λ_1 is $\max(0, -\min(\phi))$ to guarantee the positivity of the final solution.

4.2 Binless difference detection

When computing differences, we modify the likelihoods containing a signal contribution in the following way

$$\forall(i,j) \in B_b \quad \begin{cases} c_{ij1} \sim \mathrm{NB}(e^{\phi_{b\ \mathrm{ref}}}\mu_{ij\ \mathrm{ref}}, \alpha) \\ c_{ij2} \sim \mathrm{NB}(e^{\phi_{b\ \mathrm{ref}}+\delta_b}\mu_{ij}, \alpha) \end{cases} \tag{43}$$

The target is

$$\begin{aligned} \mathcal{L} = &-\frac{1}{2}\sum_b \left[\left(\frac{\hat{\phi}^{[k]}_{b\ \mathrm{ref}} - \phi_{b\ \mathrm{ref}}}{\sigma^{[k]}_{s_{b\ \mathrm{ref}}}}\right)^2 + \left(\frac{\hat{\phi}^{[k]}_b - \phi_{b\ \mathrm{ref}} - \delta_b}{\sigma^{[k]}_{s_b}}\right)^2\right] \\ &- \lambda_2 \sum_{b,b'\ \mathrm{neighbors}} |\delta_b - \delta_{b'}| - \lambda_1 \sum_b |\delta_b| \end{aligned} \tag{44}$$

We do not enforce any sign constraint on δ_b, but we enforce $\phi_{b\ \mathrm{ref}} \geqslant 0$. Therefore, at each IRLS iteration, we compute δ,

$$\phi_{b\ \mathrm{ref}} = \max\left\{\frac{\hat{\phi}^{[k]}_{b\ \mathrm{ref}}/\sigma^{[k]\,2}_{b\ \mathrm{ref}} + (\hat{\phi}^{[k]}_b - \delta_b)/\sigma^{[k]\,2}_b}{1/\sigma^{[k]\,2}_{b\ \mathrm{ref}} + 1/\sigma^{[k]\,2}_b}, 0\right\} \tag{45}$$

and finally $\hat{\phi}^{[k+1]}$ for the next IRLS iteration until convergence.

To optimize λ_2 and λ_1, we proceed as described in the previous subsection. The minimum value for λ_1 is set by the numerical tolerance imposed on the procedure.

5 Binned matrices

5.1 Available matrices

The number of count types in bin b is simply

$$\text{ncounts}_b \equiv 4\,|B_b| \tag{46}$$

The raw, or observed matrix is the sum of counts in that bin

$$\text{observed}_b \equiv \sum_{(i,j)\in B_b} c_{ij}^{\text{up}} + c_{ij}^{\text{down}} + c_{ij}^{\text{close}} + c_{ij}^{\text{far}} \tag{47}$$

The background matrix is the sum of all background means

$$\text{background}_b \equiv \sum_{(i,j)\in B_b} \sum_{t\in\text{Types}} \mu_{ij}^t = \sum_{(i,j)\in B_b} e^{e_C}(\iota_i+\rho_i)(\iota_j+\rho_j)f_{ij} \tag{48}$$

The associated standard deviation is

$$\text{background.sd}_b \equiv \left(\sum_{(i,j)\in B_b} \sum_{t\in\text{Types}} \mu_{ij}^t + \frac{{\mu_{ij}^t}^2}{\alpha}\right)^{1/2} \tag{49}$$

Note that we also sum over cut site intersections at which no ligation event was observed.
The fitted diagonal decay can be displayed in matrix form, and is

$$\text{decaymat}_b \equiv \frac{1}{\text{ncounts}_b} \sum_{(i,j)\in B_b} 4f_{ij} \tag{50}$$

The fitted biases can be aggregated and displayed in matrix form, and are

$$\text{biasmat}_b \equiv \frac{1}{\text{ncounts}_b} \sum_{(i,j)\in B_b} \iota_i + \iota_j + \rho_i + \rho_j \tag{51}$$

The residual matrix is the observed matrix divided by the expected (i.e. background+signal) matrix.
For comparison to other normalization methods, we propose "normalized" matrices. These matrices correspond to the data corrected by genomic biases and sequencing depth only. However, we strongly encourage users to work on binless matrices (see below) whenever possible.
The binless signal matrix is $\exp\phi$ put in matrix form. Note that the binless signal matrix is always greater than one. When there is no signal, the binless signal matrix equals one.

The binless matrix is the sum of the binless signal matrix and the decay matrix
The binless difference matrix is $\exp\delta$ put in matrix form. Note that the difference matrix is always positive, and when there is no significant difference, its value is 1.

5.2 Grouping

Sometimes, it can be sought to combine, for example, multiple biological replicates into a single dataset. These grouped matrices are obtained in a procedure identical to previously, with the difference that a given bin B_b now contains data from all datasets to be grouped.

6 Fast binless

6.1 Model

Normalization and interaction detection Fast binless is an approximation to the previous model, aimed at large datasets. Its input is a matrix of raw data, at the desired base resolution. Biases found on the diagonal are not used. Let c_{ijk} be the number of contacts (in all categories) observed for dataset k in bin (i, j) with $i <= j$, and n_{ijk} the number of cut site intersections in that bin. The likelihood is

$$c_{ijk} \sim \mathrm{NB}(e^{e_{C_k}} b_i b_j f_{ij} s_{ijk}, \alpha) \tag{52}$$

We model b and f using a GAM, and s using the sparse weighted fused lasso on the upper corner of a 2D square grid. For the genomic bias b_i, we assume each count c_{ijk} was observed at the center of bin i. For the decay bias f_{ij}, we assume count c_{ijk} was observed at the distance between bin centers j and i. b (and f) is centered by using the weights provided in n_{ijk}, summed along a row (resp. counter-diagonal). f is constrained to be monotonically decreasing except close to the diagonal (default 10kb).

Fast binless seeks to determine a signal matrix s_{ijk} at a fixed λ_1, λ_2 and α. It follows the same design and procedures than the exact implementation described in the previous sections, but on a binned representation of the data, and with a more efficient and scalable C++ implementation. To speed up the calculation even more, the normalization can be restricted to a maximum distance.

Difference detection For differences with respect to a reference, the model becomes

$$c_{ij1} \sim \mathrm{NB}(e^{e_{C1}} b_i b_j f_{ij} s_{ij\ \mathrm{ref}}, \alpha) \tag{53}$$

$$c_{ij2} \sim \mathrm{NB}(e^{e_{C2}} b_i b_j f_{ij} s_{ij\ \mathrm{ref}} e^{\delta_{ij}}, \alpha) \tag{54}$$

The only estimation performed is that of s_{ref} (using eq. 45) and δ using the fused lasso. All other parameters are held fixed to their optimum.

6.2 Estimation of parameters

We estimate the dispersion parameter α, the soft-thresholding λ_1 and the neighbour fusion penalty λ_2 of the fused lasso regression by normalizing selected small regions of the full matrix with optimized binless and use them for the normalization of the full matrix with fast binless. Squared submatrices of constant size are selected along the diagonal, where TADs and loops (features referred as signal for now on) are located.

λ_2 penalizes differences between neighboring values. It is the most important parameter in the estimation. Optimized binless spends most of its time optimizing that parameter. Too high values will favor the fusion of features with the surrounding contacts flattening the result and preventing its detection as signal while too low values will highlight many isolated contacts instead of grouping them as a single feature. Sup. Fig. 13 shows some examples of the variation of λ_2 of submatrices of increasing size and the corresponding signal matrix.

Optimized binless does not detect signal in some matrices spanning 400kb (left column). For these matrices, there are either no features or those are not statistically significant, and the value of λ_2 is high. When we increase the size of those matrices and features are detected, the value of λ_2 drops and remains low with larger sizes. When signal is detected in the 400kb window, the value of λ_2 remains low, even if the feature is surrounded by regions with no signal. This fact suggests that the minimum value of λ_2 in the submatrices is a good estimation for bigger matrices containing them.

The normalization of every submatrix to find the lowest value of λ_2 requires too much processing time for the procedure to be efficient. Therefore a pre-selection of submatrices is needed based on some statistics that can be calculated much faster. Different statistics (kurtosis, skewness, standard deviation, number of counts and standard deviation of the directionality index (DI)) have been evaluated to see if they are good predictors of low values of λ_2. The standard deviation of the directionality index (DI) has been chosen as a good predictor. The DI varies considerably in the positions of TAD boundaries and therefore its standard deviation will be higher in matrices containing those boundaries as compared to matrices where no boundaries exist.

For the normalization of full chromosomes, submatrices of 600Kb at 5kb base resolution (2400kb at 20kb base resolution) are extracted every 100kb from the full matrix. Regions containing features usually have higher interaction counts. Therefore, candidates with low number of counts compared to the average are discarded.

The standard deviation of the DI is then calculated in the remaining candidates. A subset of them having high values of the statistic are normalized using optimized binless. The submatrix with the lowest λ_2 is then selected to provide α and λ_2 for the normalization of the full chromosome with Fast binless. We use $\lambda_1 = 0$ for the normalization using fast binless and threshold the signal matrix a posteriori.

To calculate the value of λ_1 we consider the hypothesis corroborated by the observed matrices of the absence of signal far out the diagonal. This also corresponds to the

bet-on-sparsity principle essential for proper working of sparse fused lasso. Therefore we set the thresholding parameter λ_1 equal to the median of the signal at distances larger than 25Mb.

In Sup. Fig. 14 we present the results of the procedure by comparing the signal of submatrices selected as predictors of λ_2 and α normalized with optimized binless and the same region of the full chromosome normalized with fast binless.

Supplementary References

[1] Hastie T. and Tibshirani R. "Generalized Additive Models". In: *Stat. Sci.* 1 (3 1986), pp. 297–318 (cit. on p. 2).

[2] S.N Wood. *Generalized Additive Models: An Introduction with R*. Chapman and Hall/CRC, 2006 (cit. on pp. 2–4, 7, 9).

[3] Pya N. and Wood S. N. "Shape constrained additive models". In: *Stat. Comput.* 25 (3 2015), pp. 543–559 (cit. on p. 2).

[4] Stefan Lang and Andreas Brezger. "Bayesian P-Splines". In: *J Comp. Graph. Stat.* 13.1 (2004), pp. 183–212 (cit. on p. 3).

[5] Stefan Lang and Andreas Brezger. "Generalized structured additive regression based on Bayesian P-splines". In: *Comput. Stat. Data Anal.* 50 (2006), pp. 967–991 (cit. on p. 3).

[6] Eilers P. H. C. and Marx B. D. "Flexible Smoothing with B-splines and penalties". In: *Stat. Sci.* 11 (2 1996), pp. 89–102 (cit. on p. 3).

[7] Andrew Gelman. "Prior distributions for variance parameters in hierarchical models (comment on article by Browne and Draper)". In: *Bayesian Anal.* 1.3 (Sept. 2006), pp. 515–534. DOI: 10.1214/06-BA117A (cit. on p. 3).

[8] W Tansey and JG Scott. "A fast and flexible algorithm for the graph-fused lasso". In: (2015). arXiv: 1505.06475 (cit. on pp. 3, 10).
