# Supplementary material for: Binless normalization of Hi-C data provides significant interaction and difference detection independent of resolution
Source: Nat Commun. 2019 Apr 26;10:1938. doi: 10.1038/s41467-019-09907-2 (PMC6486590; doi:10.1038/s41467-019-09907-2)
Supplement: Supplementary file 3 — Description of Additional Supplementary Files [file 41467_2019_9907_MOESM3_ESM.pdf]

Description of Additional Supplementary Files

File Name: Supplementary Data 1
Description: Datasets, parameters, comparisons and sample sizes used in this manuscript.
